# Moving toward precision in prenatal evidence-based home visiting to achieve good birth outcomes: assessing the alignment of local programs with their national models

**DOI:** 10.1186/s12913-023-09815-8

**Published:** 2023-07-29

**Authors:** Ciara Z. Spinosa, Lori Burrell, Kelly M. Bower, Kay O’Neill, Anne K. Duggan

**Affiliations:** 1 Johns Hopkins Bloomberg School of Public Health, 615 N. Wolfe Street, Baltimore, MD 21205 USA; 2 Johns Hopkins School of Nursing, 525 N. Wolfe Street, Baltimore, MD 21205 USA

**Keywords:** Home visiting, Behavior change, Birth outcomes, Prenatal, Intervention implementation

## Abstract

**Background:**

Low birthweight and preterm birth rates are higher in the United States than in other developed countries and exhibit pronounced racial inequities. Home visiting is a strategy to promote equity in birth outcomes. Research points to precision home visiting as the path to equity. The purpose of this study is to describe local programs’ risk reduction priorities, intended behavioral pathways, and expectations of home visitors; compare these local program features with those of their national model; and assess the strength of implementation systems to support staff in meeting job expectations.

**Methods:**

We surveyed local programs implementing one of four evidence-based home visiting models that aim to promote good birth outcomes: Family Spirit, Healthy Families America, Nurse-Family Partnership, and Parents as Teachers.

**Results:**

Representatives from 169 local programs completed the survey. Overall, 59% endorsed all their model’s high priority risks, 16% endorsed all its required behavioral pathways, and 11% endorsed all its required techniques. Local programs went beyond their national model’s explicit intentions. Overall, 91% of local programs prioritized risks beyond those of their model, 85% endorsed behavioral pathways beyond those of their model, 95% endorsed visitors’ use of techniques not explicitly endorsed by their model but compatible with it, and 19% endorsed use of techniques judged incompatible by their model. Implementation system strength was positively associated with local program and model expectations.

**Conclusions:**

Precision home visiting to achieve health equity requires shared learning of what works best for whom. This observational study showed the Precision Paradigm’s usefulness for cross-model research to advance precision.

**Supplementary Information:**

The online version contains supplementary material available at 10.1186/s12913-023-09815-8.

## Background

Rates of low birthweight and preterm birth in the United States (US) are higher than in other developed countries [1] and exhibit pronounced racial inequities [2]. Maternal health behaviors associated with birth outcomes include diet, physical activity, tobacco and alcohol use, and adherence to medical regimens [3–5]. These health behaviors are themselves influenced by individual-, family-, and environmental-level contextual factors.

Home visiting is a service strategy to improve and promote equity in health and socioeconomic outcomes, including birth outcomes. Federally-funded scale up of evidence-based home visiting in the US began in 2010 via the Maternal, Infant and Early Childhood Home Visiting (MIECHV) Program [6]. The MIECHV Program recognizes 20 home visiting models as evidence-based [7]; models vary in features such as intended outcomes, family eligibility, curricula, and intended dosage [8].

Local home visiting programs are uniquely positioned to promote equity in health and socio-economic outcomes. They typically target families and communities for which contextual factors adversely affect these outcomes. Home visitors can come to understand how contextual factors influence health and parenting because they interact with families in the home setting. They can use this understanding to optimize benefits for highly diverse families through evidence-based tailoring – that is, precision – matched with families’ assets, needs, and preferences. Such precision is the path to equity.

Existing research confirms the need for precision home visiting but does not inform how to optimize benefits across diverse families and communities. Average effects for full home visiting models are persistently small [8–10]. To illustrate, the national evaluation of the MIECHV Program found no significant effects on focal outcomes such as prenatal behaviors, birth outcomes, or health care use in the first year of life [10].

The literature confirms the diversity of families and communities served [8], but gives little actionable insight on why effects vary among population subgroups. There are two main reasons. First, research typically considers full home visiting models as “the intervention”, rather than considering home visiting models as complex service strategies comprised of many interventions. Second, research typically has tested moderation only in post hoc analyses and only for easily defined subgroups, such as defined by race, rather than by using study designs to test theory-based, a priori hypotheses regarding moderators.

To advance home visiting efficiency and return on investment, the field must increase average effect sizes. This could be done two ways – by targeting only the subgroups who currently benefit from home visiting, or by optimizing impacts through evidence-based tailoring to extend benefits beyond these subgroups. Both strategies require knowing which interventions within home visiting work best, for whom, in which contexts. Such knowledge can be gained through impact research that: focuses on interventions within and across full models; incorporates mediators, moderators, and intervention reach and engagement; uses a standard framework and terminology to promote cross-study learning; and is hypothesis-driven with a rationale supported by theory and empirical research [11].

There is broad and growing emphasis on specificity in intervention design and testing. Behavior change science emphasizes specifying how interventions are designed and how and why they are expected to promote target behaviors as mediators of outcomes [12]. Several researchers have developed ways to specify the ‘core components’ of interventions [13], and at least one approach to organizing core components by level of abstraction, from principles to contextual and structural elements, to the intervention practices and techniques used by providers [14].

Precision home visiting, that is, evidence-based tailoring of services to optimize benefits, requires going beyond the traditional focus of evidence-based model accreditation. Accreditation typically focuses on local program adherence to the national model’s principles, contextual elements, and structural elements. In contrast, precision home visiting considers national model – local program alignment on the target behaviors as mediators of intended outcomes and on intervention practices and techniques to promote target behaviors. Evidence-based models’ required behavioral pathways and intervention techniques are the reference for assessing alignment, which has two aspects – adherence and enhancement. We define *adherence* as local programs’ endorsement of the target behaviors and intervention techniques required by their national model. We define *enhancement* as the extent to which local programs endorse target behaviors and intervention techniques beyond those required by their national model. These two aspects have parallels in broad initiatives to distinguish home visiting core components from enhancements. Further, they are relevant for the design and testing of cross-model enhancements, as illustrated in current research testing a cardiovascular health promotion intervention [15]. In addition, national model—local program alignment on target behaviors and intervention techniques might be an important influence on the adequacy of systems to support staff in meeting job expectations.

**Fig. 1:**
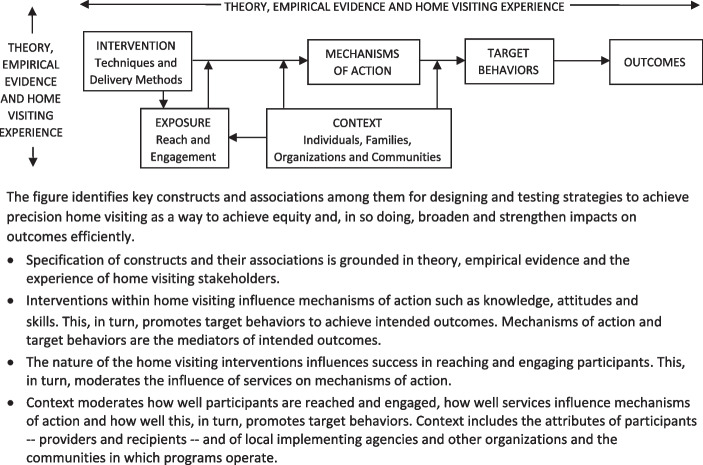
Home Visiting Precision Paradigm (adapted from the Human Behaviour Change Project). The figure identifies key constructs and associations among them for designing and testing strategies to achieve precision home visiting as a way to achieve equity and, in so doing, broaden and strengthen impacts on outcomes efficiently. Specification of constructs and their associations is grounded in theory, empirical evidence and the experience of home visiting stakeholders. Interventions within home visiting influence mechanisms of action such as knowledge, attitudes and skills. This, in turn, promotes target behaviors to achieve intended outcomes. Mechanisms of action and target behaviors are the mediators of intended outcomes. The nature of the home visiting interventions influences success in reaching and engaging participants. This, in turn, moderates the influence of services on mechanisms of action. Context moderates how well participants are reached and engaged, how well services influence mechanisms of action and how well this, in turn, promotes target behaviors. Context includes the attributes of participants – providers and recipients – and of local implementing agencies and other organizations and the communities in which programs operate. (Human Behaviour Change Project. 2022. Available from: https://www.humanbehaviourchange.org/)

The Home Visiting Applied Research Collaborative (HARC) is a national platform for scientific collaboration and innovative research to strengthen home visiting’s contribution to health equity by optimizing impacts across diverse maternal and child health populations. We are building the Precision Paradigm (Fig. 1) to support research toward precision [16]. The goal is an ontology – a standardized framework and terminology for defining interventions, mediators, and moderators at a granular level – to accelerate the generation, generalizability, and actionability of new knowledge across home visiting models and studies [17]. We are building on others’ work [18–21] and using consensus-building methods [22] to specify each part of the new paradigm to assure its relevance and usefulness for home visiting.

The Precision Paradigm defines seven concepts and the relationships among them to help research collaborators see clearly how aspects of interventions within home visiting are expected to improve a specific outcome in a specific context. The framework highlights how *theory, evidence, and home visiting experience* can guide our understanding of how home visiting interventions achieve outcomes and allows users to specify how change is theoretically intended to occur. It can be used to identify and specify: an intended home visiting *outcome*, such as full term birth; the relevant *target behaviors* needed to achieve that outcome, such as stopping or reducing smoking; the *mechanisms of action,* or causal processes through which an intervention is thought to influence these behaviors, such as intervention recipients' beliefs about the consequences of smoking; and key aspects of the *intervention*, which include specific intervention techniques, such as providing information about the health consequences of smoking for the fetus, details about how the intervention is delivered, such as the intended schedule, mode and source, and tailoring via intentional variation in intervention content and/or delivery based on characteristics of the recipient and the setting. It also considers factors that might influence the path from intervention to outcomes in important ways. For example, how aspects of *usage*, which includes reach and engagement, and *contextual factors*, such as recipient attributes (e.g. primary language, readiness to change) and community characteristics (e.g. culture, resources), might influence an intervention’s effectiveness.

In earlier work, we demonstrated the Precision Paradigm’s utility for describing national models’ priorities for risk reduction, intended behavioral pathways to good birth outcomes, and stance on home visitors’ use of specific intervention techniques to promote families’ progress on those pathways [23]. The current paper builds on that work; it assesses alignment by comparing local programs’ risk reduction priorities, intended behavioral pathways, and expectations of home visitors with those of their national model. It also assesses the strength of systems to support home visitors in meeting job expectations.

## Methods

This study used data from an earlier survey of evidence-based home visiting models and newly collected data of local programs implementing those models. The methods for the model survey have been described elsewhere [23]. Study methods and results are reported following the Strengthening the Reporting of Observational Studies in Epidemiology (STROBE) Statement for cross-sectional studies [24].

### Sample

We used non-probability sampling of local programs implementing one of four US-based evidence-based home visiting models that identified promotion of good birth outcomes as a central focus: Family Spirit (FS), Healthy Families America (HFA), Nurse-Family Partnership (NFP), and Parents as Teachers (PAT) [23]. We recruited local programs in two ways. We enlisted the help of the four national models to invite and encourage their local affiliates to participate. We also invited local programs in HARC’s practice-based research network to participate. Recruitment materials specified that only one staff member serving in a leadership role at their program should complete the survey.

Representatives from 169 local programs across 43 states and territories completed the survey. Most were program directors or managers (51%) or supervisors (41%) and nearly all had served in their role for over two years (85%). The greatest proportion of local programs completing the survey implemented PAT (42%), followed by HFA (34%), FS (12%), and NFP (11%). Most local programs served rural communities (73%), and nearly half served urban (47%) and suburban (40%) communities. Three quarters of the local programs employed ≤ 10 home visitors and about half served ≤ 80 families.

### Data collection

The online survey assessed four aspects of local program design: 1) priorities for reducing risks for poor birth outcomes, 2) intentions to promote target behaviors to reduce priority risks, 3) expectations for home visitors' use of specific intervention techniques to promote target behaviors, and 4) implementation system supports for home visitors' use of expected intervention techniques. The survey was developed for this study and is described below (Additional file 1). The survey link was emailed to local program site representatives and was available December 2020 to March 2021. The Johns Hopkins Bloomberg School of Public Health Institutional Review Board determined the study was not human subjects research.

### Measurement

#### Local programs’ stance on risk factors, behaviors, behavioral pathways, and intervention techniques

##### Risk reduction priorities

The survey focused on ten common, modifiable, evidence-based [3–5, 25] risks for low birth weight and premature birth that could be reduced through home visiting during the current pregnancy***.*** The risks fell into four groups: 1) biologic (infection, diabetes, high blood pressure); 2) psychosocial well-being (high stress, depression, intimate partner violence); 3) behavioral health (tobacco use, alcohol use, illicit drug use); and 4) health care use (inadequate prenatal care). Respondents rated the priority their local program gave to reducing each risk. Response choices were: *not a priority*, *low priority*, *moderate priority*, *high priority*, and *not sure*. A *priority risk* was defined as one whose reduction was rated as low, moderate, or high priority.

##### Target behaviors

Drawing from the literature cited above, the survey focused on 14 behaviors that could be promoted via home visiting to reduce one or more of the focal risks in pregnancy. The behaviors fell into four conceptual groups: 1) lifestyle (physical activity, healthy diet, stress reduction activities, use of social supports); 2) health (self-monitor physiologic indicators, adhere to prescribed medication regimen, use condoms, develop a safety plan); 3) behavioral health (stop or reduce tobacco use, stop or reduce alcohol use, stop or reduce illicit drug use); and 4) health care use (adhere to prenatal care visit schedule, alert prenatal care provider to warning signs, engage in substance use treatment). Respondents rated expectations of home visitors for promoting each behavior to reduce each of the program’s priority risks. Response choices were *required*, *recommended but not required*, *no expectation but compatible with our program*, *not compatible with our program*, and *not sure*.

##### Intended behavioral pathways

The 10 risk factors and 14 behaviors together defined 41 pathways to good birth outcomes (Table 1). An *intended pathway* for a local program was defined as one linking a recommended or required target behavior with a priority risk.

##### Endorsed intervention techniques

Respondents rated their programs' stance regarding home visitors' use of each of 23 intervention technique categories (Additional file 2) to promote healthy behaviors to reduce risks for poor birth outcomes. We created the categories by adapting an existing taxonomy [18] and adding referral and coordination techniques, which are commonly used in home visiting but were not in the existing taxonomy. Response choices were *required*, *recommended but not required*, *no expectation but compatible with our program*, *not compatible with our program*, and *not sure*. Although we focused on technique *categories*, we refer to them as ‘techniques’ in this report. An *endorsed technique* is one that the local program either required or recommended home visitors to use.

**Table 1:** Potential pathways to promote good birth outcomes^a^

	**Biologic Risks**			**Psychological Risks**			**Behavioral Health Risks**			**Health Care Risk**
**Target Behaviors**	**High Blood Pressure**	**Diabetes**	**Infection** ^**b**^	**High Stress**	**Depression**	**IPV** ^**c**^	**Tobacco Use**	**Alcohol Use**	**Illicit SU** ^**d**^	**Inadequate PNC** ^**e**^
**Lifestyle**										
Engage in physical activity	X	X		X	X					
Adhere to a healthy diet	X	X								
Engage in stress reduction activities				X	X	X	X	X	X	
Use social supports				X	X	X	X	X	X	
**Health Behaviors**										
Self-monitor physiologic indicators	X	X								
Adhere to medication regimen	X	X	X		X		X		X	
Use condoms			X							
Develop a safety plan						X				
**Behavioral Health**										
Stop or reduce tobacco use	X						X			
Stop or reduce alcohol use	X							X		
Stop or reduce illicit SU									X	
**Health Care Use**										
Adhere to PNC visit schedule	X	X	X		X					X
Alert PNC provider to warning signs	X	X			X					
Engage in SU treatment									X	

^a^Each X represents a unique pathway to good birth outcomes by promoting a specific target behavior to reduce a specific risk contributing to poor birth outcomes. There are 41 pathways; ^b^Sexually transmitted, vaginal, or urinary tract; ^c^Intimate partner violence; ^d^Substance use (heroin or cocaine); ^e^Inadequate prenatal care is defined as late entry or inadequate number of visits post enrollment in HV

#### Local programs’ alignment with their national model

##### Risk reduction priorities

We defined three levels of local program adherence to the model’s high priority risks: local program high prioritization of all such risks; local program prioritization (low, moderate, or high) of all such risks but not high prioritization of all; and local program prioritization (low, moderate, or high) of some but not all such risks.

We defined two indicators of local program enhancement to the model: prioritization of at least one risk beyond their model’s priority risks; and high prioritization of at least one such risk.

##### Behavioral pathways

We defined three levels of local program adherence to the model’s required behavioral pathways: local program requirement of all such pathways; local program requirement or recommendation of all such pathways but not requirement of all; and local program requirement or recommendation of some but not all such pathways.

We defined two indicators of local program enhancement to the model: local program requirement or recommendation of at least one pathway beyond their model’s intended (required or recommended) behavioral pathways; and local program requirement of at least one such pathway.

##### Intervention techniques

We defined three levels of local program adherence to the model’s required techniques: local program requirement of all such techniques; local program requirement or recommendation of all such techniques but not requirement of all; and local program requirement or recommendation of some but not all such techniques.

We defined two indicators of local program enhancement to the model: local program requirement or recommendation of at least one technique beyond their model’s endorsed techniques; and local program requirement of at least one such technique.

#### Local programs’ implementation systems

Respondents rated the implementation system for each endorsed technique in terms of six components identified in the literature: written policy, training, assessment, supervisory support, peer support, and monitoring and feedback [26–28]. Response choices were: *fully in place*; *partially in place*; *not in place;* and *not sure*. We calculated an implementation system score for each endorsed technique as the sum of scores for the six components. We allocated one point if the component was fully in place, a half point if the component was partially in place, and no points if the component was not in place or the respondent was unsure if it was in place. Thus, the *implementation system score* for an endorsed technique could range from 0 (no component partially in place) to 6 (all components fully in place).

#### Model stance on risk priorities, behavioral pathways, and endorsed techniques

In the project’s first phase, national model representatives completed surveys similar to the local program survey described above to rate their model’s stance on the same risks, behaviors, behavioral pathways, and intervention techniques as in the local program survey [23].

Model stance on *risk priorities* and *behavioral pathways* was defined as described above for local programs. Model stance on *intervention techniques* was measured at a more granular level than for local programs. While the local program survey elicited the local program’s stance on home visitors’ use of each technique to promote target behaviors *in general*, the model survey elicited the model’s stance on home visitors’ use of each technique in each of its intended behavioral pathways. We used the models’ pathway-specific ratings to create a four-category general rating of techniques: 1) model required the technique for at least one intended pathway; 2) model never required the technique but recommended the technique for at least one intended pathway; 3) model never required or recommended the technique but rated the technique as not expected but compatible for at least one intended pathway; 4) model rated the technique as incompatible for all intended pathways.

### Analysis

#### Description of local programs

We used descriptive statistics to characterize local programs’ risk reduction priorities, intended behavioral pathways, endorsed techniques, and implementation system scores. Local program was the unit of analysis.

#### Local programs’ alignment with their national model

We used descriptive statistics to characterize local programs’ adherence to and enhancement of their national model’s risk reduction priorities, intended behavioral pathways and endorsed techniques.

#### Association of local program and model expectations with implementation system strength

We used multiple linear regression to test whether and how local program and model expectations for home visitors’ use of specific techniques were associated with local programs’ implementation system scores. Implementation strength scores were only derived for techniques that a local program rated as recommended or required. For this analysis, we used an analytic file where technique, rather than local program, was the unit of analysis. Local program expectation for technique use was included as a binary variable (required vs. not required) and model expectation was included as a categorical variable (required, recommended, not expected but compatible, and not compatible, with not compatible included as the reference).

## Results

### Local programs’ prioritization of risks

Local programs prioritized reducing 9.5 risks on average (SD = 1.2; range 4–10). Levels of priority varied by type of risk (Table 2). Nearly all local programs gave a high priority to reducing psychosocial and health care use risks, most gave a high priority to reducing behavioral health risks, and a third did so for reducing biologic risks. It was rare for a local program not to prioritize reducing psychosocial, behavioral health, and health care use risks, but about one in eight did not prioritize reducing biologic risks.

**Table 2:** Local programs’ stance on risk prioritization (*n* = 169)

Risk Factors for Poor Birth Outcomes	Not a Priority	Low Priority	Moderate Priority	High Priority
**Biologic Risks**				
High Blood Pressure	12%	18%	34%	35%
Diabetes	11%	18%	36%	36%
Infection	12%	18%	36%	34%
**Psychosocial Risks**				
Intimate Partner Violence	1%	2%	3%	94%
High Stress	0%	1%	8%	92%
Depression	0%	1%	2%	97%
**Behavioral Health Risks**				
Tobacco Use	2%	4%	24%	70%
Alcohol Use	2%	4%	21%	73%
Illicit Drug Use	2%	4%	14%	79%
**Health Care Use Risks**				
Inadequate Prenatal Care	< 1%	2%	11%	87%

Percentages may not add to 100 due to rounding. Local programs responding “Not Sure” were excluded from the denominator. The percentage of local programs responding “Not Sure” ranged from 0%-2% across the risks

### Local program – Model alignment on priority risks

#### Adherence

Nearly three-fifths (59%) of local programs gave a high priority to all of their model’s high priority risks (Table 3). An additional third of local programs prioritized all such risks but did not rate all as a high priority. Few programs, only 5%, rated some but not all such risks as a priority. Overall, 87% of local programs prioritized all of their model’s priority risks.

**Table 3:** Local program adherence to and enhancement of national model’s prioritized risks (*n* = 169)

	% of Local Programs
**Adherence to National Model’s High Priority Risks**	
Local program rates all such risks as high priority	59%
Local program rates all such risks as a priority, but does not rate all a high priority	36%
Local program rates some but not all such risks as a priority	5%
**Enhancement to National Model’s Prioritized Risks**^a^	
Local program rates at least one risk as a priority	91%
Local program rates at least one risk as a high priority	36%

^a^Local program prioritization of risks that their national model rated not a priority. One model rated at least one risk as not a priority, analysis limited to local programs implementing that model

#### Enhancement

Nearly all local programs (91%) prioritized risks beyond those of their model. Over a third gave a high priority to at least one such risk.

### Local programs’ intended behavioral pathways

Local programs varied greatly in their number of intended pathways (M = 34, SD = 8.8; range: 5–41). All 41 pathways were designated as intended – either required OR recommended – by over half of local programs (Table 4, first number in each pair). Eighteen pathways were so designated by > 90% of local programs (dark grey shading). Twelve pathways – all related to biologic risk – were designated as intended by < 75% of local programs (no shading).

**Table 4:**
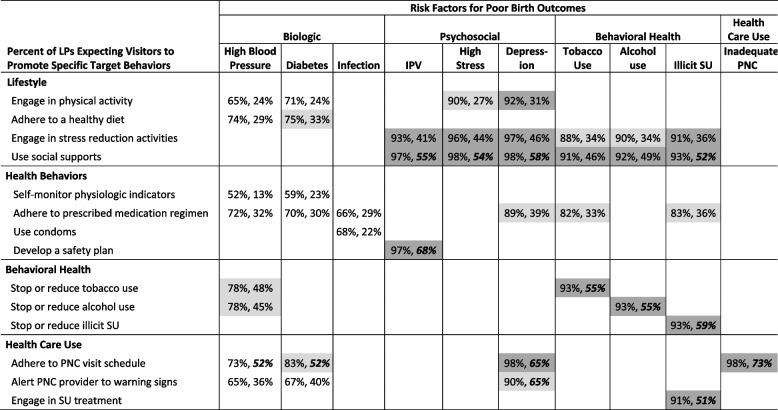
Local programs’ stance on intended and required behavioral pathways to good birth outcomes^a^ (*n* = 169)

^a^Within each cell, the first number represents the percent of LPs that designate the pathway as intended, meaning they recommend or require that home visitors promote the target behavior (row) to reduce the priority risk (column). The second number represents the percent of LPs that ‘REQUIRE’ that home visitors promote the target behavior to reduce the priority risk. Local programs responding “Not Sure” were excluded from the denominator. The percentage of local programs responding “Not Sure” ranged from 0%-3% across the pathways

KEY: Shading – No Shading – <75% of programs designate pathway as intended;  – 75-90% of programs designate pathway as intended;  – >90% of programs designate pathway as intended. **Bolding** – Bolded percentages represent the pathways required by >50% of LPs

Local programs designated an average of 17 behavioral pathways as required and varied greatly in this (SD = 13.5; range 0–41). Fourteen pathways were required by over half of local programs (Table 4, italicized second number in each pair). These included most pathways involving social supports; developing a safety plan; all pathways involving adherence to prenatal care visit schedules; and pathways involving behavioral health and health care use to reduce psychological and behavioral health risk factors.

### Local program – Model alignment on intended behavioral pathways

#### Adherence

Few local programs, only 16%, required all of their model’s required behavioral pathways (Table 5). All other local programs were partially adherent to their model’s required pathways. About half designated some required pathways as recommended rather than required. About a third required or recommended some, but not all, of the model’s required pathways.

**Table 5:** Local program adherence to and enhancement of national model’s behavioral pathways (*n* = 169)

	% of Local Programs
**Adherence to National Model’s Required Behavioral Pathways**^a^	
Local program requires all such pathways	16%
Local program either recommends or requires all such pathways, but does not require all	52%
Local program requires or recommends some but not all such pathways	32%
**Enhancement to National Model’s Intended Behavioral Pathways**^b^	
Local program requires or recommends at least one pathway	85%
Local program requires at least one pathway	62%

^a^One model required one or more pathways, analysis limited to local programs implementing that model; ^b^Local program designation of behavioral pathways as intended (required or recommended) that their national model rated not expected but compatible. Three models rated one or more pathways as not expected but compatible, analysis limited to local programs implementing those models

#### Enhancement

Most local programs had intended behavioral pathways beyond those of their model. Nearly two-thirds of local programs designated at least one such pathway as required.

### Local programs’ endorsement of intervention techniques

Local programs endorsed an average of 16.8 techniques (SD 5.0; range 2–23). The proportion of local programs endorsing a technique varied by technique, from 11–99% **(**Table 6). Over 80% of local programs required the techniques ‘Referral and Linkage’ and ‘Monitoring and Follow-up of Referral’ while 50–78% of local programs required the techniques ‘Goals and Planning,’ ‘Provide Social Support,’ ‘Monitoring and Feedback,’ ‘Suggest or Arrange Social Support,’ and ‘Coordination with Other Services’. Fewer than 25% of local programs required ‘Mental Regulation,’ ‘Antecedents,’ ‘Self-Identity,’ ‘Identity as Example to Others,’ and ‘Scheduled Consequences.’ Sixteen techniques were rated not compatible by some local programs. ‘Scheduled Consequences,’ was rated not compatible by 77% of local programs.

**Table 6:** Local programs’ expectations regarding the use of intervention technique categories (*n* = 169)

	Local Program Stance on Technique Category			
Technique Category	Not Compatible with Program	No Expectation, but Compatible with Program	Endorsed	
			Recommended	Required
Referral and Linkage	0%	1%	12%	87%
Monitoring and Follow-up of Referral	0%	2%	13%	85%
Goals and Planning	0%	3%	20%	77%
Provide Social Support	1%	1%	22%	76%
Monitoring and Feedback	1%	10%	33%	57%
Suggest or Arrange Social Support	0%	4%	41%	55%
Coordination with Other Services	0%	7%	38%	55%
Credible Source	0%	16%	35%	49%
Assess Readiness for Change	1%	21%	31%	47%
Self-Belief	1%	19%	44%	37%
Shape Knowledge of Behavior	1%	20%	43%	35%
Natural Consequences	4%	30%	34%	32%
Behavior Observation	1%	23%	46%	31%
Incentives and Rewards	8%	27%	36%	29%
Comparison of Outcomes	0%	20%	52%	27%
Associations to Promote Wanted Behavior	4%	28%	43%	26%
Repetition and Substitution	2%	29%	44%	25%
Associations to Deter Unwanted Behavior	7%	30%	39%	25%
Mental Regulation	8%	27%	41%	24%
Antecedents	4%	31%	41%	24%
Self-Identity	4%	41%	37%	18%
Identity as Example to Others	8%	44%	37%	11%
Scheduled Consequences	77%	13%	8%	3%

Percentages may not add to 100 due to rounding. Local programs responding “Not Sure” were excluded from the denominator. The percentage of local programs responding “Not Sure” ranged from 0%-9% across the techniques

### Local program – Model alignment on intervention techniques

#### Adherence

Few local programs, only 11%, required all of their model’s required intervention techniques (Table 7). All other local programs were partially adherent to their model’s required intervention techniques. About a third designated some required techniques as recommended rather than required. About half required or recommended some, but not all, of their model’s required techniques.

**Table 7:** Local program adherence to and enhancement of national model’s endorsed techniques

	% of Local Programs
**Adherence to National Model’s Required Techniques**^a^	
Local program requires all such techniques	11%
Local program either recommends or requires all such techniques, but does not require all	37%
Local program requires or recommends some but not all such techniques	52%
**Enhancement to National Model’s Endorsed Techniques**	
Techniques Rated Not Expected but Compatible by Model^b^	
Local program requires or recommends at least one such technique	95%
Local program requires at least one such technique	65%
Techniques Rated Not Compatible by Model^c^	
Local program requires or recommends at least one such technique	19%
Local program requires at least one such technique	7%

^a^Three models required at least one technique for at least one intended pathway, analysis limited to local programs implementing those models (*n* = 111); ^b^Two models rated at least one technique as not expected but compatible across all of their intended pathways, analysis limited to local programs implementing those models (*n* = 129); ^c^Three models rated at least one technique as not compatible across all of their intended pathways, analysis limited to local programs implementing those models (*n* = 98)

#### Enhancement

Nearly all local programs required or recommended that home visitors use intervention techniques beyond those their model did not expect visitors to use, but that it felt were compatible with the model. Nearly two-thirds of local programs required use of one or more such techniques.

A fifth of local programs required or recommended that home visitors use techniques rated incompatible by the model, and a few, 7%, required using such techniques.

### Local programs’ implementation system scores

Implementation system scores varied greatly by technique. Mean scores ranged from 2.1 to 5.0 and the percent of local programs with a perfect score for any given technique ranged from 12 to 44% (Table 8). Local programs requiring a technique were significantly more likely than those recommending it to have a complete implementation system for the majority of techniques. Even so, fewer than half of local programs requiring a technique had a perfect implementation system score, even when the model also required the technique.

**Table 8:** Local Program (LP) technique implementation system scores (*n* = 169)

	Implementation System Score^b^ (Mean (sd))	Percent of LPs with Complete Implementation Systems^c^		
			LP Stance on Technique	
Technique^a^	Overall	Overall	Recommend	Require
*Techniques Required by* > *50% of LPs*				
Referral and Linkage	5.0 (1.2)	44%	38%	45%*
Monitoring and Follow-up of Referral	4.6 (1.6)	40%	25%	42%
Goals and Planning	4.9 (1.3)	42%	24%	47%*
Provide Social Support	4.2 (1.6)	27%	14%	30%
Monitoring and Feedback	4.2 (1.8)	30%	18%	36%*
Suggest or Arrange Social Support	3.9 (1.8)	24%	12%	32%*
Coordination with Other Services	4.4 (1.7)	36%	34%	38%
*Techniques Required by 25–50% of LPS*				
Credible Source	4.1 (1.8)	30%	13%	42%*
Assess Readiness for Change	3.3 (1.9)	17%	5%	25%*
Self-Belief	3.4 (2.1)	20%	10%	32%
Shape Knowledge of Behavior	3.3 (1.9)	20%	9%	34%*
Natural Consequences	3.1 (2.1)	18%	3%	35%*
Behavior Observation	3.3 (2.0)	24%	18%	33%*
Incentives and Rewards	3.5 (2.0)	23%	17%	31%
Comparison of Outcomes	3.1 (2.0)	19%	12%	32%*
Associations to Promote Wanted Behavior	3.1 (2.1)	20%	11%	34%*
Repetition and Substitution	2.9 (2.0)	16%	6%	32%*
Associations to Deter Unwanted Behavior	2.6 (2.0)	12%	6%	21%
*Techniques Required by* < *25% of LPS*				
Mental Regulation	3.4 (2.0)	17%	13%	23%
Antecedents	2.9 (2.0)	16%	9%	29%*
Self-Identity	2.8 (2.1)	17%	13%	24%
Identity as Example to Others	2.6 (2.0)	12%	12%	10%
Scheduled Consequences	2.1 (2.3)	18%	12%	34%

^*^p < .05; ^a^Listed in descending order by percent of local programs requiring the technique; ^b^Possible implementation system component scores range from 0–6 with higher scores indicating stronger implementation systems; ^c^The percent of local programs with an implementation system component score of 6, indicating all six implementation system components are fully in-place

### Association of local programs’ implementation system scores with model stance on techniques

**Fig. 2:**
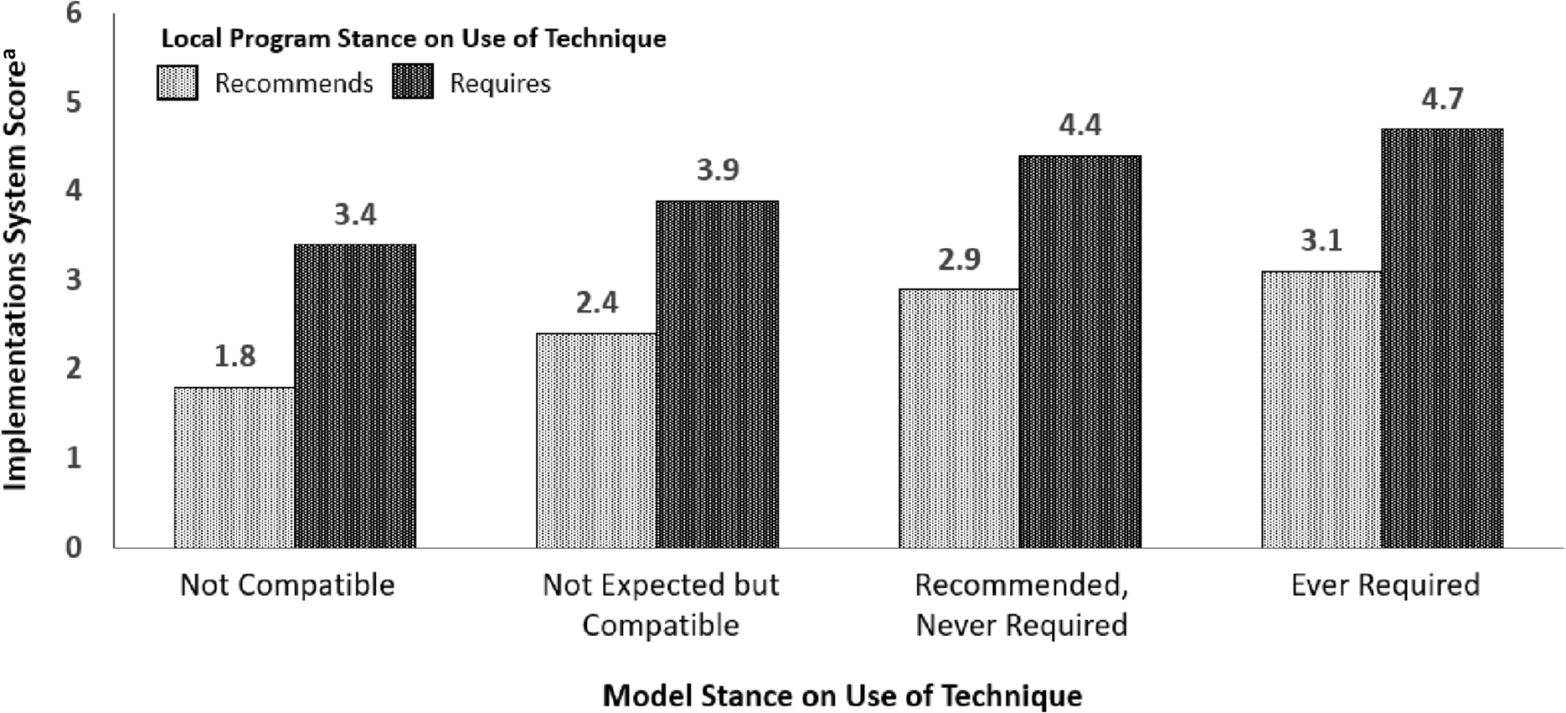
Predicted Implementations System Scores by Local Program and Model Stance on Techniques. (Techniques are the unit of analysis. The analysis includes techniques that local programs rated as recommended or required (*n* = 2843). ^a^Possible implementation system scores range from 0–6 with higher scores indicating stronger implementation systems

Implementation system scores were positively associated with the local program’s and the model’s stance on use of a technique (Fig. 2). Implementation system scores for supporting the use of specific techniques were significantly higher for techniques required by local programs compared to those they recommended (β = 1.53, *p* < 0.001). Compared to techniques that models rated as not compatible, implementation system scores were significantly higher for techniques that models rated as recommended (β = 1.10, *p* = 0.01) and required (β = 1.31, *p* = 0.01). There was not a significant difference in implementation system scores for techniques that models required compared to those recommended (β = 0.21, *p* = 0.13).

## Discussion

Continued investment in and support of home visiting requires evidence of overall effectiveness in improving intended outcomes and in reducing inequities in outcomes. Home visiting programs serve highly diverse families and communities; research points to precision home visiting as the path to equity through evidence-based tailoring of interventions. But efforts to achieve precision must be grounded in a solid understanding of how current interventions within home visiting are defined, the intended mediating links from interventions to outcomes, the anticipated moderators of these links, and the adequacy of current systems to support staff in providing interventions competently. Building on this understanding, the field can improve existing interventions and support systems to increase efficiency and the return on investment made possible through improved outcome equity.

The Mother and Infant Home Visiting Program Evaluation (MIHOPE) is one study that began to build this understanding. MIHOPE used standardized methods across four evidence-based models – HFA, NFP, PAT and Early Head Start – to define outcome priorities, expectations of home visitors, and implementation systems to support home visitors to meet job expectations. It assessed alignment of local programs with their national models on a few features, such as frequency of supervision. Thus, MIHOPE implementation study results [8] are useful context for interpreting results of the current study. Besides MIHOPE, we are unaware of other published reports examining what we have in this study.

The current study is narrower than MIHOPE because it focused on birth outcomes. It is similar to MIHOPE because it used parallel methods to measure and compare the perspectives of national models and local programs. It went farther than MIHOPE by using a typology of intervention techniques to describe expectations of home visitors; by focusing on behavioral pathways as mediators of birth outcomes; and by assessing current implementation systems for specific intervention techniques.

Like MIHOPE, our study found that local programs had ambitious aims. Many local programs prioritized reducing all or nearly all risks, intended to promote family progress on nearly all behavioral pathways, and endorsed home visitors’ use of nearly all techniques. Our study went beyond MIHOPE by assessing local program – model alignment on expectations for visitors’ use of intervention techniques and on intended behavioral pathways. We confirmed the need to assess rather than assume adherence, as only 59% of local programs endorsed all of their model’s high priority risks, only 16% endorsed all of their model’s required behavioral pathways, and only 11% endorsed all of its required techniques.

Like MIHOPE, our study showed that local programs often enhanced the evidence-based models they implemented. For example, 91% of local programs prioritized risks beyond those prioritized by their model, 85% endorsed behavioral pathways beyond those of their model, 95% endorsed visitors’ use of techniques compatible with but not explicitly endorsed by their model, and 19% endorsed use of techniques judged incompatible by their model.

As in MIHOPE, we found that implementation system strength was positively associated with local and model expectations. Like MIHOPE, we found great opportunity to strengthen implementation systems, with fewer than half of local programs having a perfect implementation system score for techniques required by both the local program and the model.

### Methodologic considerations

This cross-model study demonstrated the feasibility and usefulness of the Precision Paradigm as a framework for research to broaden and strengthen home visiting impacts. The paradigm allowed national models and local programs to specify intended behavioral pathways and intervention techniques in a standardized way so that it was possible to assess their alignment. Such specification is a necessary foundation for assessing clarity, commonalities, and differentiation of approaches. Such assessments are necessary for comparative effectiveness studies of what works best for whom.

There are many ways to improve on our methods. We relied on the literature to identify focal risks, behavioral pathways, and techniques; it will be important going forward to tap other sources as well, including family perspectives. We grouped individual techniques into categories to reduce data collection burden; this might have masked meaningful differences in expectations across techniques within category. We did not ascertain local programs’ accreditation status and so could not test that as a moderator of alignment. We focused on only two aspects of the Precision Paradigm; future work should consider other aspects and the links among them. We assessed only the views of local program leadership; it is important to compare views across staff within local program. Furthermore, we did not examine whether respondents answered all questions with equal attention and accuracy or the degree to which social acceptability bias may have played a role. Our rudimentary assessment of implementation systems could be strengthened by applying more sophisticated tools to archival documents as data sources.

While this study identified substantial departures of local programs from their national model it did not address the reasons for departures. One likely contributor to lack of adherence is that local programs are unaware of national models’ stance on behavioral pathways and intervention techniques. National models tend to focus on philosophical principles and structural elements rather than intervention practices in defining their core components for accreditation. Local program enhancements could also arise from local programs’ misunderstanding of national models’ requirements. Another contributor to local program enhancements is the influence of external forces beyond national models, such as funders. MIHOPE’s implementation study found evidence of this; while MIECHV Program funding typically made up a minority of a local program’s support, it expanded the role of home visitors, with over half of home visitors in MIHOPE reporting that their responsibilities were greater since their program began to receive MIECHV funding. In short, the current study identified widespread departures of local programs from their national models, but future research is needed to understand and address the reasons and consequences of such departures.

Lastly, the study did not assess actual service delivery. Research comparing intended and actual service delivery is critically important, as is research comparing service delivery as reported by providers and as assessed through visit observation. Theory posits that providers are more likely to meet job expectations if expectations are clear; we need empirical research to test this assumption. But MIHOPE’s implementation study revealed varied alignment of visit content as logged by home visitors and as observed in a sample of video recorded visits [8]. This speaks to the importance of designing management information systems that promote accurate and complete reporting of service delivery in alignment with intended intervention techniques. This need becomes more pressing as the field moves toward greater reliance on cross-model collaborative research using existing data [29].

### Implications

The need for clearly defined and prioritized precision services to promote good birth outcomes is even clearer when one considers that families complete an average of only eight home visits prenatally [10]. It is not realistic to expect home visitors to address a broad range of behavioral pathways using a loosely defined set of techniques. Rather, they must be efficient in ascertaining family assets, needs, concerns and preferences, and skilled in choosing from an array of acceptable techniques and delivery methods to focus on the pathways most likely to promote good outcomes in light of context.

We found that many local programs deviated from their evidence-based models. Local programs often opted not to adhere to the high priorities and requirements of their evidence-based models. Yet, they often enhanced their national model by prioritizing risks, pursuing behavioral pathways, and endorsing visitors’ use of techniques beyond their evidence-based model’s priorities, intended pathways and endorsed techniques. We also found that implementation systems often fell short in supporting staff to use required intervention techniques, and that there were opportunities to strengthen these systems even for techniques required by both the local program and the evidence-based model.

The field would benefit from understanding the causes and consequences of local programs’ deviations from their evidence-based models and gaps in their implementation systems. It might be inconsequential when local programs pursue pathways beyond those of their model. On the other hand, it might detract from fidelity in ways that attenuate benefits for families. Our results provide a starting point for scientific collaborations to determine how best to strengthen local program – model alignment and implementation systems.

While this study focused on prenatal home visiting to promote good birth outcomes, its methods could be used to compare local programs and evidence-based models’ approaches to promote other outcomes. While the study focused on services for families, its methods could be applied to systems-level interventions to address structural and systemic determinants of health, and to implementation system interventions to promote staff competence and service quality.

## Conclusions

Precision home visiting to achieve health equity requires shared learning of what works best for whom. Each group with an interest in home visiting plays a critical role in such research. This study demonstrated the Precision Paradigm’s usefulness for cross-model research to advance precision. It confirmed the home visiting field’s ambitious expectations of front-line staff. It highlighted opportunities to strengthen the alignment of local programs with their evidence-based models and to strengthen implementation systems to support staff in meeting job expectations. As the field shifts toward actionable research using the Precision Paradigm, it can build a strong foundation for innovation to advance precision in services, service systems and implementation systems and, in so doing, strengthen average effects, efficiency, and return on investment.

## Supplementary Information

**Additional file 1.** 

**Additional file 2.** 

## Data Availability

The datasets generated and analyzed during the current study are not publicly available due to the proprietary nature of the national home visiting models but are available from the corresponding author on reasonable request.

## Abbreviations

HARC: Home Visiting Applied Research Collaborative
HFA: Healthy Families America
MIECHV Program: Maternal, Infant and Early Childhood Home Visiting Program
NFP: Nurse-Family Partnership
PAT: Parents as Teachers
FS: Family Spirit
