# Supplementary material for: Moving toward precision in prenatal evidence-based home visiting to achieve good birth outcomes: assessing the alignment of local programs with their national models
Source: BMC Health Serv Res. 2023 Jul 29;23:812. doi: 10.1186/s12913-023-09815-8 (PMC10386274; doi:10.1186/s12913-023-09815-8)
Supplement: Supplementary file 1 — Additional file 1. [file 12913_2023_9815_MOESM1_ESM.pdf]

Cross-Model Precision Prenatal HV Project Survey for Local Programs

Overview

Thank you for taking part in the Cross-Model Precision Prenatal Home Visiting Study. The study objective is to describe techniques endorsed by local programs implementing evidence-based home visiting models to reduce preterm birth and low birth weight.

The purpose of this survey is to understand your program's:

1. Priorities for reducing each of 10 risk factors for preterm birth and low birth weight
2. Expectations of home visitors for promoting each of 14 health behaviors to reduce one or more risk factors
3. Views on home visitors' use of each of 23 techniques to promote healthy behaviors among pregnant women to reduce poor birth outcomes
4. Current implementation systems to support home visitors in their use of techniques

<u>Only one person from your program should complete this survey</u>. This survey should take about 1 hour to complete. We will provide $50 remuneration for completing the survey.

You can finish the survey in more than one session if you'd like. You can exit the survey by closing the webpage. There is no 'save' button to press. Your answers are saved automatically each time you exit. If you leave and return later, be sure you enter using the <u>same email link</u>, <u>same computer</u> and <u>same browser</u>. The link will open the survey where you left off.

Responding to this survey request is voluntary; it is your choice. Completing this survey and submitting it to us means you consent to participate in the study. You may choose not to answer any question that we ask.

Confidentiality

Researchers at Johns Hopkins University (JHU) will collect and analyze the data. Your answers will be kept confidential. When you complete this survey your name and contact information will be temporarily visible to select members of the research team at JHU but will then be replaced with a confidential ID number. Your name and contact information will be stored separately from your ID number on a secure server. The research team at JHU will not share your name or any other identifying information about you to anyone. Results will be reported only in the aggregate, in such a way that the information you provide will not be identifiable.

Contact Us

If you have <u>any questions</u>, including uncertainty about a particular term or phrase, or problems while taking the survey, please contact Ciara Zagaja at czagaja1@jhu.edu or (202) 643-8302.

SECTION A: BACKGROUND
In this section, you will be asked background information about your home visiting program and your role with the program.

A01. Please indicate all home visiting models your program implements.

- ☐ Family Spirit
- ☐ Healthy Families America
- ☐ Nurse Family Partnership
- ☐ Parents as Teachers
- ☐ None of the above [SKIP to end of survey]

A02. [DISPLAY IF MORE THAN 1 MODEL SELECTED IN A01] Of the models that your program implements, which model are you most familiar with?

Note: Please answer the remaining survey questions with **only this one** model in mind.

- ☐ Family Spirit
- ☐ Healthy Families America
- ☐ Nurse Family Partnership
- ☐ Parents as Teachers

A03. Where is your program located? (state) [Dropdown]

A04. What types of community does your program serve? Check all that apply.

- ☐ Urban
- ☐ Suburban
- ☐ Rural
- ☐ Frontier

A05. How many home visitors does your program employ (full-time and part-time)?

- ☐ 1-4
- ☐ 5-10
- ☐ 11-15
- ☐ 16-20
- ☐ More than 20
- ☐ Not sure

A06. How many families are currently enrolled in your program?

- ☐ 0-20
- ☐ 21-40
- ☐ 41-60
- ☐ 61-80
- ☐ More than 80
- ☐ Not sure

A07. What is your role at your program?

- ☐ Clinical director
- ☐ Program manager
- ☐ Supervisor
- ☐ Other (please specify): [Text box] ______________________________

A08. How many years have you served in this current role? IF MORE THAN 6 MONTHS ROUND TO THE NEAREST WHOLE NUMBER OF YEARS.

- ☐ Less than 6 months
- ☐ ___ years [Text box] __________________________________

SECTION B: PROGRAM PRIORITIES AND EXPECTATIONS
This section will ask about your program's priorities for addressing 10 risk factors for preterm birth and low birth weight and its expectations of home visitors for promoting 14 behaviors to reduce one or more risk factors.

B01. In your program, how high a priority is it to address each of the following risk factors to reduce poor birth outcomes (preterm birth or low birth weight) for pregnant women?

	Not a priority	Low priority	Moderate priority	High priority	Not sure
High blood pressure	☐	☐	☐	☐	☐
Diabetes	☐	☐	☐	☐	☐
Infection – sexually transmitted, vaginal, or urinary tract	☐	☐	☐	☐	☐
Intimate partner violence	☐	☐	☐	☐	☐
High stress	☐	☐	☐	☐	☐
Depression	☐	☐	☐	☐	☐
Tobacco use	☐	☐	☐	☐	☐
Alcohol use	☐	☐	☐	☐	☐
Illicit drug use – heroin or cocaine	☐	☐	☐	☐	☐
Inadequate prenatal care – late entry or inadequate number of visits post enrollment in HV	☐	☐	☐	☐	☐

B02. [For each question below, DISPLAY THE QUESTION **ONLY IF** the response for the corresponding risk factor in B01 is low, moderate, or high priority.]

a. What is your program's expectation of home visitors for promoting each behavior below to reduce the risk of poor birth outcomes (low birthweight or preterm birth) **for pregnant women with high blood pressure**?

Note: Check 'Required' if your program expects home visitors to promote a behavior with ANY subset of pregnant women with high blood pressure. Your program does not have to expect visitors to promote it with all pregnant women with high blood pressure to check 'Required'.

	Required	Recommended, but not required	No expectation, but compatible with our program	Not compatible with our program	Not sure
Engage in physical activity	☐	☐	☐	☐	☐
Adhere to a healthy diet	☐	☐	☐	☐	☐
Stop or reduce tobacco use	☐	☐	☐	☐	☐
Stop or reduce alcohol use	☐	☐	☐	☐	☐
Adhere to prescribed medication regimen	☐	☐	☐	☐	☐
Self-monitor blood pressure	☐	☐	☐	☐	☐
Adhere to prenatal care visit schedule	☐	☐	☐	☐	☐
Alert prenatal care provider to warning signs of uncontrolled blood pressure	☐	☐	☐	☐	☐

b. What is your program's expectation of home visitors for promoting each behavior below to reduce the risk of poor birth outcomes (low birthweight or preterm birth) **for pregnant women with diabetes**?

Note: Check 'Required' if your program expects home visitors to promote a behavior with ANY subset of pregnant women with diabetes. Your program does not have to expect visitors to promote it with all pregnant women with diabetes to check 'Required'.

	Required	Recommended, but not required	No expectation, but compatible with our program	Not compatible with our program	Not sure
Engage in physical activity	☐	☐	☐	☐	☐
Adhere to a healthy diet	☐	☐	☐	☐	☐
Adhere to prescribed medication regimen	☐	☐	☐	☐	☐

Self-monitor blood glucose	☐	☐	☐	☐	☐
Adhere to prenatal care visit schedule	☐	☐	☐	☐	☐
Alert prenatal care provider to warning signs of uncontrolled diabetes	☐	☐	☐	☐	☐

c. What is your program's expectation of home visitors for promoting each behavior below to reduce the risk of poor birth outcomes (low birthweight or preterm birth) **for pregnant women at risk for infection (sexually transmitted, vaginal, or urinary tract)**?

Note: Check 'Required' if your program expects home visitors to promote a behavior with ANY subset of pregnant women at risk for infection. Your program does not have to expect visitors to promote it with all pregnant women at risk for infection to check 'Required'.

	Required	Recommended, but not required	No expectation, but compatible with our program	Not compatible with our program	Not sure
Use condoms	☐	☐	☐	☐	☐
Adhere to prescribed medication regimen	☐	☐	☐	☐	☐

d. What is your program's expectation of home visitors for promoting each behavior below to reduce the risk of poor birth outcomes (low birthweight or preterm birth) **for pregnant women experiencing intimate partner violence**?

Note: Check 'Required' if your program expects home visitors to promote a behavior with ANY subset of pregnant women experiencing intimate partner violence. Your program does not have to expect visitors to promote it with all pregnant women experiencing intimate partner violence to check 'Required'.

	Required	Recommended, but not required	No expectation, but compatible with our program	Not compatible with our program	Not sure
Engage in stress reduction activities (such as meditation, mindfulness, breathing exercises, journaling, and performing an enjoyed activity)	☐	☐	☐	☐	☐

Use social supports (such as seeking support from colleagues, friends, family, or support groups)	☐	☐	☐	☐	☐
Develop a safety plan	☐	☐	☐	☐	☐

e. What is your program's expectation of home visitors for promoting each behavior below to reduce the risk of poor birth outcomes (low birthweight or preterm birth) **for pregnant women with high stress**?

Note: Check 'Required' if your program expects home visitors to promote a behavior with ANY subset of pregnant women with high stress. Your program does not have to expect visitors to promote it with all pregnant women with high stress to check 'Required'.

	Required	Recommended, but not required	No expectation, but compatible with our program	Not compatible with our program	Not sure
Engage in stress reduction activities (such as meditation, mindfulness, breathing exercises, journaling, and performing an enjoyed activity)	☐	☐	☐	☐	☐
Engage in physical activity	☐	☐	☐	☐	☐
Use social supports (such as seeking support from colleagues, friends, family, or support groups)	☐	☐	☐	☐	☐

f. What is your program's expectation of home visitors for promoting each behavior below to reduce the risk of poor birth outcomes (low birthweight or preterm birth) **for pregnant women with depression**?

Note: Check 'Required' if your program expects home visitors to promote a behavior with ANY subset of pregnant women with depression. Your program does not have to expect visitors to promote it with all pregnant women with depression to check 'Required'.

	Required	Recommended, but not required	No expectation, but compatible	Not compatible with our program	Not sure

			with our program		
Engage in stress reduction activities (such as meditation, mindfulness, breathing exercises, journaling, and performing an enjoyed activity)	☐	☐	☐	☐	☐
Engage in physical activity	☐	☐	☐	☐	☐
Use social supports (such as seeking support from colleagues, friends, family, or support groups)	☐	☐	☐	☐	☐
Adhere to prescribed medication regimen	☐	☐	☐	☐	☐
Adhere to prenatal care visit schedule	☐	☐	☐	☐	☐
Alert prenatal care provider to warning signs of harm to self or others	☐	☐	☐	☐	☐

g. What is your program's expectation of home visitors for promoting each behavior below to reduce the risk of poor birth outcomes (low birthweight or preterm birth) **for pregnant women who use tobacco**?

Note: Check 'Required' if your program expects home visitors to promote a behavior with ANY subset of pregnant women who use tobacco. Your program does not have to expect visitors to promote it with all pregnant women who use tobacco to check 'Required'.

	Required	Recommended, but not required	No expectation, but compatible with our program	Not compatible with our program	Not sure
Engage in stress reduction activities (such as meditation, mindfulness, breathing exercises, journaling, and performing an enjoyed activity)	☐	☐	☐	☐	☐
Adhere to prescribed medication regimen (such as smoking cessation aids)	☐	☐	☐	☐	☐
Use social supports (such as seeking support from	☐	☐	☐	☐	☐

colleagues, friends, family, or support groups)					
Stop or reduce tobacco use	☐	☐	☐	☐	☐

h. What is your program's expectation of home visitors for promoting each behavior below to reduce the risk of poor birth outcomes (low birthweight or preterm birth) **for pregnant women who use alcohol**?

<u>Note</u>: Check 'Required' if your program expects home visitors to promote a behavior with <u>ANY</u> subset of pregnant women who use alcohol. Your program does not have to expect visitors to promote it with all pregnant women who use alcohol to check 'Required'.

	Required	Recommended, but not required	No expectation, but compatible with our program	Not compatible with our program	Not sure
Engage in stress reduction activities (such as meditation, mindfulness, breathing exercises, journaling, and performing an enjoyed activity)	☐	☐	☐	☐	☐
Use social supports (such as seeking support from colleagues, friends, family, or support groups)	☐	☐	☐	☐	☐
Stop or reduce alcohol use	☐	☐	☐	☐	☐

i. What is your program's expectation of home visitors for promoting each behavior below to reduce the risk of poor birth outcomes (low birthweight or preterm birth) **for pregnant women who use illicit drugs (heroin or cocaine)**?

<u>Note</u>: Check 'Required' if your program expects home visitors to promote a behavior with <u>ANY</u> subset of pregnant women who use illicit drugs. Your program does not have to expect visitors to promote it with all pregnant women who use illicit drugs to check 'Required'.

	Required	Recommended, but not required	No expectation, but compatible with our program	Not compatible with our program	Not sure

Engage in stress reduction activities (such as meditation, mindfulness, breathing exercises, journaling, and performing an enjoyed activity)	☐	☐	☐	☐	☐
Adhere to prescribed medication regimen	☐	☐	☐	☐	☐
Use social supports (such as seeking support from colleagues, friends, family, or support groups)	☐	☐	☐	☐	☐
Stop or reduce illicit drug use	☐	☐	☐	☐	☐
Engage in substance use treatment	☐	☐	☐	☐	☐

j. What is your program's expectation of home visitors for promoting the behavior below to reduce the risk of poor birth outcomes (low birthweight or preterm birth) **for pregnant women with inadequate prenatal care (late entry or inadequate number of visits post enrollment in HV)**?

Note: Check 'Required' if your program expects home visitors to promote a behavior with ANY subset of pregnant women with inadequate prenatal care. Your program does not have to expect visitors to promote it with all pregnant women with inadequate prenatal care to check 'Required'.

	Required	Recommended, but not required	No expectation, but compatible with our program	Not compatible with our program	Not sure
Adhere to prenatal care visit schedule	☐	☐	☐	☐	☐

SECTION C: TECHNIQUES

Home visitors might use many different techniques to promote behaviors (such as those asked about in the previous section) that will help a pregnant woman reduce risks for poor birth outcomes. We would like to understand your program's expectations of home visitors for using each of 23 different techniques.

The 23 techniques asked about in this survey are primarily behavior modification techniques. This list is not exhaustive and may not include all of the techniques your program would like home visitors to use.

C01. A) Technique #1: "**Assess Readiness for Change**"

Definition: Gather information about the pregnant woman's readiness to change a behavior.

Please indicate your program's expectation of home visitors' use of "**Assess Readiness for Change**" to promote pregnant women to engage in healthy behaviors <u>in order to reduce risk factors for low birthweight and preterm birth.</u>

- ☐ We **require** home visitors to use this technique with at least some pregnant women
- ☐ We **recommend** that home visitors use this technique with at least some pregnant women, but we do not require them to use it with any
- ☐ We **neither require nor recommend** home visitors to use this technique, but it is compatible with our program
- ☐ We **do not want home visitors to use this technique** because it is not compatible with our program
- ☐ Not sure

B) Technique #2: "**Goals & Planning**"

Definition: Assist the pregnant woman to: set a behavior change goal; develop a plan to meet the goal using strategies to overcome barriers and increase facilitators; review her progress toward the goal; modify the goal or plan as needed.

Please indicate your program's expectation of home visitors' use of "**Goals & Planning**" to promote pregnant women to engage in healthy behaviors <u>in order to reduce risk factors for low birthweight and preterm birth.</u>

<u>Note</u>: This technique includes multiple strategies. If your program has different levels of expectation in using strategies within the technique, select the response that reflects the highest level of expectation among the strategies.

- ☐ We **require** home visitors to use this technique with at least some pregnant women
- ☐ We **recommend** that home visitors use this technique with at least some pregnant women, but we do not require them to use it with any
- ☐ We **neither require nor recommend** home visitors to use this technique, but it is compatible with our program

- ☐ We **do not want home visitors to use this technique** because it is not compatible with our program
- ☐ Not sure

C) Technique #3: "**Monitoring & Feedback**"

Definition: Monitor the pregnant woman's progress in changing a behavior; give feedback on that progress; establish ways for the pregnant woman to self-monitor her progress.

Please indicate your program's expectation of home visitors' use of "**Monitoring & Feedback**" to promote pregnant women to engage in healthy behaviors <u>in order to reduce risk factors for low birthweight and preterm birth.</u>

<u>Note</u>: This technique includes multiple strategies. If your program has different levels of expectation in using strategies within the technique, select the response that reflects the highest level of expectation among the strategies.

- ☐ We **require** home visitors to use this technique with at least some pregnant women
- ☐ We **recommend** that home visitors use this technique with at least some pregnant women, but we do not require them to use it with any
- ☐ We **neither require nor recommend** home visitors to use this technique, but it is compatible with our program
- ☐ We **do not want home visitors to use this technique** because it is not compatible with our program
- ☐ Not sure

D) Technique #4: "**Provide Social Support**"

Definition: Directly provide the pregnant woman encouragement, emotional support or practical help to perform a behavior.

Please indicate your program's expectation of home visitors' use of "**Provide Social Support**" to promote pregnant women to engage in healthy behaviors <u>in order to reduce risk factors for low birthweight and preterm birth.</u>

- ☐ We **require** home visitors to use this technique with at least some pregnant women
- ☐ We **recommend** that home visitors use this technique with at least some pregnant women, but we do not require them to use it with any
- ☐ We **neither require nor recommend** home visitors to use this technique, but it is compatible with our program
- ☐ We **do not want home visitors to use this technique** because it is not compatible with our program
- ☐ Not sure

E) Technique #5: "**Suggest or Arrange Social Support**"

Definition: Suggest or assist the pregnant woman to seek encouragement, emotional support or practical help to perform a behavior from a friend, relative, colleague, or group.

Please indicate your program's expectation of home visitors' use of "**Suggest or Arrange Social Support**" to promote pregnant women to engage in healthy behaviors <u>in order to reduce risk factors for low birthweight and preterm birth.</u>

- ☐ We **require** home visitors to use this technique with at least some pregnant women
- ☐ We **recommend** that home visitors use this technique with at least some pregnant women, but we do not require them to use it with any
- ☐ We **neither require nor recommend** home visitors to use this technique, but it is compatible with our program
- ☐ We **do not want home visitors to use this technique** because it is not compatible with our program
- ☐ Not sure

F) Technique #6: "**Natural Consequences**"

Definition: Provide written, verbal or visual information about a behavior's health, emotional, social or environmental consequences; encourage her to assess her feelings after attempts to perform a wanted behavior; raise her awareness of future regret about performing an unwanted behavior.

Please indicate your program's expectation of home visitors' use of "**Natural Consequences**" to promote pregnant women to engage in healthy behaviors <u>in order to reduce risk factors for low birthweight and preterm birth.</u>

<u>Note</u>: This technique includes multiple strategies. If your program has different levels of expectation in using strategies within the technique, select the response that reflects the highest level of expectation among the strategies.

- ☐ We **require** home visitors to use this technique with at least some pregnant women
- ☐ We **recommend** that home visitors use this technique with at least some pregnant women, but we do not require them to use it with any
- ☐ We **neither require nor recommend** home visitors to use this technique, but it is compatible with our program
- ☐ We **do not want home visitors to use this technique** because it is not compatible with our program
- ☐ Not sure

G) Technique #7: "**Shape Knowledge of Behavior**"

Definition: Provide information or instruction to shape the pregnant woman's knowledge of how to perform a behavior. This includes identification of behavioral 'triggers' and their perceived causes. 'Triggers' are thoughts or situations that lead to performance of the unwanted behavior.

Please indicate your program's expectation of home visitors' use of "**Shape Knowledge of Behavior**" to promote pregnant women to engage in healthy behaviors in order to reduce risk factors for low birthweight and preterm birth.

- ☐ We **require** home visitors to use this technique with at least some pregnant women
- ☐ We **recommend** that home visitors use this technique with at least some pregnant women, but we do not require them to use it with any
- ☐ We **neither require nor recommend** home visitors to use this technique, but it is compatible with our program
- ☐ We **do not want home visitors to use this technique** because it is not compatible with our program
- ☐ Not sure

H) Technique #8: "**Antecedents**"

Definition: Change or support change of the pregnant woman's physical or social surroundings to facilitate performing a wanted behavior, create barriers to an unwanted behavior, or avoid cues to an unwanted behavior.

Please indicate your program's expectation of home visitors' use of "**Antecedents**" to promote pregnant women to engage in healthy behaviors in order to reduce risk factors for low birthweight and preterm birth.

Note: This technique includes multiple strategies. If your program has different levels of expectation in using strategies within the technique, select the response that reflects the highest level of expectation among the strategies.

- ☐ We **require** home visitors to use this technique with at least some pregnant women
- ☐ We **recommend** that home visitors use this technique with at least some pregnant women, but we do not require them to use it with any
- ☐ We **neither require nor recommend** home visitors to use this technique, but it is compatible with our program
- ☐ We **do not want home visitors to use this technique** because it is not compatible with our program
- ☐ Not sure

I) Technique #9: "**Behavior Observation**"

Definition: Demonstrate a behavior; provide an observable example of a behavior; draw attention to others' performance of a behavior as a model.

Please indicate your program's expectation of home visitors' use of "**Behavior Observation**" to promote pregnant women to engage in healthy behaviors in order to reduce risk factors for low birthweight and preterm birth.

Note: This technique includes multiple strategies. If your program has different levels of expectation in using strategies within the technique, select the response that reflects the highest level of expectation among the strategies.

- ☐ We **require** home visitors to use this technique with at least some pregnant women
- ☐ We **recommend** that home visitors use this technique with at least some pregnant women, but we do not require them to use it with any
- ☐ We **neither require nor recommend** home visitors to use this technique, but it is compatible with our program
- ☐ We **do not want home visitors to use this technique** because it is not compatible with our program
- ☐ Not sure

J) Technique #10: "**Associations to Promote Wanted Behavior**"

Definition: Identify, introduce, or alter social or environmental prompts or cues to promote a wanted behavior.

Please indicate your program's expectation of home visitors' use of "**Associations to Promote Wanted Behavior**" to promote pregnant women to engage in healthy behaviors in order to reduce risk factors for low birthweight and preterm birth.

- ☐ We **require** home visitors to use this technique with at least some pregnant women
- ☐ We **recommend** that home visitors use this technique with at least some pregnant women, but we do not require them to use it with any
- ☐ We **neither require nor recommend** home visitors to use this technique, but it is compatible with our program
- ☐ We **do not want home visitors to use this technique** because it is not compatible with our program
- ☐ Not sure

K) Technique #11: "**Associations to Deter Unwanted Behavior**"

Definition: Identify, alter, or remove social or environmental prompts or cues to deter an unwanted behavior.

Please indicate your program's expectation of home visitors' use of "**Associations to Deter Unwanted Behavior**" to promote pregnant women to engage in healthy behaviors in order to reduce risk factors for low birthweight and preterm birth.

- ☐ We **require** home visitors to use this technique with at least some pregnant women
- ☐ We **recommend** that home visitors use this technique with at least some pregnant women, but we do not require them to use it with any
- ☐ We **neither require nor recommend** home visitors to use this technique, but it is compatible with our program
- ☐ We **do not want home visitors to use this technique** because it is not compatible with our program

- ☐ Not sure

L) Technique #12: "**Repetition & Substitution**"

Definition: Encourage the pregnant woman to practice performing a wanted behavior or substitute it for an unwanted behavior.

Please indicate your program's expectation of home visitors' use of "**Repetition & Substitution**" to promote pregnant women to engage in healthy behaviors <u>in order to reduce risk factors for low birthweight and preterm birth.</u>

- ☐ We **require** home visitors to use this technique with at least some pregnant women
- ☐ We **recommend** that home visitors use this technique with at least some pregnant women, but we do not require them to use it with any
- ☐ We **neither require nor recommend** home visitors to use this technique, but it is compatible with our program
- ☐ We **do not want home visitors to use this technique** because it is not compatible with our program
- ☐ Not sure

M) Technique #13: "**Comparison of Outcomes**"

Definition: Encourage the pregnant woman to compare the pros and cons of changing a behavior, or to compare the outcomes of changing versus not changing a behavior. Includes encouraging the pregnant woman's imagination or observation of either the consequences of an unwanted behavior or rewards for a wanted behavior.

Please indicate your program's expectation of home visitors' use of "**Comparison of Outcomes**" to promote pregnant women to engage in healthy behaviors <u>in order to reduce risk factors for low birthweight and preterm birth.</u>

- ☐ We **require** home visitors to use this technique with at least some pregnant women
- ☐ We **recommend** that home visitors use this technique with at least some pregnant women, but we do not require them to use it with any
- ☐ We **neither require nor recommend** home visitors to use this technique, but it is compatible with our program
- ☐ We **do not want home visitors to use this technique** because it is not compatible with our program
- ☐ Not sure

N) Technique #14: "**Credible Source**"

Definition: Present verbal or visual communication from a credible source in favor of or against a behavior.

Please indicate your program's expectation of home visitors' use of "**Credible Source**" to promote pregnant women to engage in healthy behaviors <u>in order to reduce risk factors for low birthweight and preterm birth.</u>

- ☐ We **require** home visitors to use this technique with at least some pregnant women
- ☐ We **recommend** that home visitors use this technique with at least some pregnant women, but we do not require them to use it with any
- ☐ We **neither require nor recommend** home visitors to use this technique, but it is compatible with our program
- ☐ We **do not want home visitors to use this technique** because it is not compatible with our program
- ☐ Not sure

O) Technique #15: "**Incentives & Rewards**"

Definition: Provide or arrange for the pregnant woman to receive a material incentive or reward (something of value) or a social incentive or reward (words of congratulation), or removal of an unpleasant consequence for making progress in performing a behavior. Includes encouraging the pregnant woman to use self-incentives or self-rewards.

Please indicate your program's expectation of home visitors' use of "**Incentives & Rewards**" to promote pregnant women to engage in healthy behaviors <u>in order to reduce risk factors for low birthweight and preterm birth.</u>

- ☐ We **require** home visitors to use this technique with at least some pregnant women
- ☐ We **recommend** that home visitors use this technique with at least some pregnant women, but we do not require them to use it with any
- ☐ We **neither require nor recommend** home visitors to use this technique, but it is compatible with our program
- ☐ We **do not want home visitors to use this technique** because it is not compatible with our program
- ☐ Not sure

P) Technique #16: "**Scheduled Consequences**"

Definition: Use a threat of future punishment or removal of a reward as a consequence of performance of an unwanted behavior; arrange for a negative consequence or punishment following performance of an unwanted behavior.

Please indicate your program's expectation of home visitors' use of "**Scheduled Consequences**" to promote pregnant women to engage in healthy behaviors <u>in order to reduce risk factors for low birthweight and preterm birth.</u>

<u>Note</u>: This technique includes multiple strategies. If your program has different levels of expectation in using strategies within the technique, select the response that reflects the highest level of expectation among the strategies.

- ☐ We **require** home visitors to use this technique with at least some pregnant women

- ☐ We **recommend** that home visitors use this technique with at least some pregnant women, but we do not require them to use it with any
- ☐ We **neither require nor recommend** home visitors to use this technique, but it is compatible with our program
- ☐ We **do not want home visitors to use this technique** because it is not compatible with our program
- ☐ Not sure

Q) Technique #17: “**Mental Regulation**”

Definition: Suggest strategies to minimize demands on the pregnant woman’s mental resources to make it easier for her to perform a wanted behavior.

Please indicate your program’s expectation of home visitors’ use of “**Mental Regulation**” to promote pregnant women to engage in healthy behaviors <u>in order to reduce risk factors for low birthweight and preterm birth.</u>

- ☐ We **require** home visitors to use this technique with at least some pregnant women
- ☐ We **recommend** that home visitors use this technique with at least some pregnant women, but we do not require them to use it with any
- ☐ We **neither require nor recommend** home visitors to use this technique, but it is compatible with our program
- ☐ We **do not want home visitors to use this technique** because it is not compatible with our program
- ☐ Not sure

R) Technique #18: “**Identity as Example to Others**”

Definition: Suggest to the pregnant woman that performing a wanted behavior might serve as an example to others.

Please indicate your program’s expectation of home visitors’ use of “**Identity as Example to Others**” to promote pregnant women to engage in healthy behaviors <u>in order to reduce risk factors for low birthweight and preterm birth.</u>

- ☐ We **require** home visitors to use this technique with at least some pregnant women
- ☐ We **recommend** that home visitors use this technique with at least some pregnant women, but we do not require them to use it with any
- ☐ We **neither require nor recommend** home visitors to use this technique, but it is compatible with our program
- ☐ We **do not want home visitors to use this technique** because it is not compatible with our program
- ☐ Not sure

S) Technique #19: “**Self-identity**”

Definition: Assist the pregnant woman to identify discrepancies between her behavior and her values or self-image; encourage her to self-identify as someone who used to perform an unwanted behavior; suggest her adopting a new perspective to change thoughts or emotions about a behavior.

Please indicate your program's expectation of home visitors' use of "**Self-identity**" to promote pregnant women to engage in healthy behaviors <u>in order to reduce risk factors for low birthweight and preterm birth.</u>

<u>Note</u>: This technique includes multiple strategies. If your program has different levels of expectation in using strategies within the technique, select the response that reflects the highest level of expectation among the strategies.

- ☐ We **require** home visitors to use this technique with at least some pregnant women
- ☐ We **recommend** that home visitors use this technique with at least some pregnant women, but we do not require them to use it with any
- ☐ We **neither require nor recommend** home visitors to use this technique, but it is compatible with our program
- ☐ We **do not want home visitors to use this technique** because it is not compatible with our program
- ☐ Not sure

T) Technique #20: "**Self-belief**"

Definition: Promote the pregnant woman's self-belief that she can successfully perform a behavior, for example by persuading her about her capabilities and encouraging her to mentally rehearse success, focus on past success or use positive self-talk.

Please indicate your program's expectation of home visitors' use of "**Self-belief**" to promote pregnant women to engage in healthy behaviors <u>in order to reduce risk factors for low birthweight and preterm birth.</u>

- ☐ We **require** home visitors to use this technique with at least some pregnant women
- ☐ We **recommend** that home visitors use this technique with at least some pregnant women, but we do not require them to use it with any
- ☐ We **neither require nor recommend** home visitors to use this technique, but it is compatible with our program
- ☐ We **do not want home visitors to use this technique** because it is not compatible with our program
- ☐ Not sure

U) Technique #21: "**Referral & Linkage**"

Definition: Provide referral or information to link the pregnant woman to a community resource to assist in performing a behavior; review progress in completing the referral; support connections in completing the referral or perform an interagency case review.

Please indicate your program's expectation of home visitors' use of "**Referral & Linkage**" to promote pregnant women to engage in healthy behaviors in order to reduce risk factors for low birthweight and preterm birth.

Note: This technique includes multiple strategies. If your program has different levels of expectation in using strategies within the technique, select the response that reflects the highest level of expectation among the strategies.

- ☐ We **require** home visitors to use this technique with at least some pregnant women
- ☐ We **recommend** that home visitors use this technique with at least some pregnant women, but we do not require them to use it with any
- ☐ We **neither require nor recommend** home visitors to use this technique, but it is compatible with our program
- ☐ We **do not want home visitors to use this technique** because it is not compatible with our program
- ☐ Not sure

V) Technique #22: "**Monitoring & Follow-up of Referral**"

Definition: Review the pregnant woman's experience accessing community resources to help her perform a behavior; assist in overcoming barriers to completing a referral.

Please indicate your program's expectation of home visitors' use of "**Monitoring & Follow-up of Referral**" to promote pregnant women to engage in healthy behaviors in order to reduce risk factors for low birthweight and preterm birth.

Note: This technique includes multiple strategies. If your program has different levels of expectation in using strategies within the technique, select the response that reflects the highest level of expectation among the strategies.

- ☐ We **require** home visitors to use this technique with at least some pregnant women
- ☐ We **recommend** that home visitors use this technique with at least some pregnant women, but we do not require them to use it with any
- ☐ We **neither require nor recommend** home visitors to use this technique, but it is compatible with our program
- ☐ We **do not want home visitors to use this technique** because it is not compatible with our program
- ☐ Not sure

W) Technique #23: "**Coordination with Other Services**"

Definition: Ask about and act on the pregnant woman's ideas on how to assist her in adhering to guidance from other providers regarding performing a behavior.

Please indicate your program's expectation of home visitors' use of "**Coordination with Other Services**" to promote pregnant women to engage in healthy behaviors in order to reduce risk factors for low birthweight and preterm birth.

- ☐ We **require** home visitors to use this technique with at least some pregnant women
- ☐ We **recommend** that home visitors use this technique with at least some pregnant women, but we do not require them to use it with any
- ☐ We **neither require nor recommend** home visitors to use this technique, but it is compatible with our program
- ☐ We **do not want home visitors to use this technique** because it is not compatible with our program
- ☐ Not sure

C02. A) [DISPLAY IF 'Required' or 'Recommend' selected in C01-A]

Please indicate whether your program has any of the following implementation systems in place to support home visitors to use the technique "**Assess Readiness for Change**"

Definition: Gather information about the pregnant woman's readiness to change a behavior.

	**Yes, Fully in Place**	**Yes, Partially in Place**	**No**	**Not sure**
1. Formal written policy clearly **defines when and how** to use the technique	☐	☐	☐	☐
2. Home visitors receive **formal training on when and how** to use the technique effectively	☐	☐	☐	☐
3. Home visitors receive **a formal assessment after completing training** to confirm they can use the technique effectively	☐	☐	☐	☐
4. ***Supervisors*** **support and encourage** home visitors to use the technique	☐	☐	☐	☐
5. ***Home visitors*** **support and encourage** one another to use the technique	☐	☐	☐	☐
6. Supervisors or other program staff **formally monitor and give feedback** on how well home visitors use the technique relative to program expectations	☐	☐	☐	☐

B) [DISPLAY IF 'Required' or 'Recommend' selected in C01-B]

Please indicate whether your program has any of the following implementation systems in place to support home visitors to use the technique "**Goals & Planning**"

Definition: Assist the pregnant woman to: set a behavior change goal; develop a plan to meet the goal using strategies to overcome barriers and increase facilitators; review her progress toward the goal; modify the goal or plan as needed.

Note: This technique includes multiple strategies. If your program has different levels of implementation systems for strategies within the technique, select the response that reflects the highest level among the strategies.

	Yes, Fully in Place	Yes, Partially in Place	No	Not sure
1. Formal written policy clearly **defines when and how** to use the technique	☐	☐	☐	☐
2. Home visitors receive **formal training on when and how** to use the technique effectively	☐	☐	☐	☐
3. Home visitors receive **a formal assessment after completing training** to confirm they can use the technique effectively	☐	☐	☐	☐
4. ***Supervisors*** **support and encourage** home visitors to use the technique	☐	☐	☐	☐
5. ***Home visitors*** **support and encourage** one another to use the technique	☐	☐	☐	☐
6. Supervisors or other program staff **formally monitor and give feedback** on how well home visitors use the technique relative to program expectations	☐	☐	☐	☐

C) [DISPLAY IF 'Required' or 'Recommend' selected in C01-C]

Please indicate whether your program has any of the following implementation systems in place to support home visitors to use the technique "**Monitoring & Feedback**"

Definition: Monitor the pregnant woman's progress in changing a behavior; give feedback on that progress; establish ways for the pregnant woman to self-monitor her progress.

Note: This technique includes multiple strategies. If your program has different levels of implementation systems for strategies within the technique, select the response that reflects the highest level among the strategies.

	Yes, Fully in Place	Yes, Partially in Place	No	Not sure
1. Formal written policy clearly **defines when and how** to use the technique	☐	☐	☐	☐
2. Home visitors receive **formal training on when and how** to use the technique effectively	☐	☐	☐	☐
3. Home visitors receive **a formal assessment after completing training** to confirm they can use the technique effectively	☐	☐	☐	☐
4. ***Supervisors*** **support and encourage** home visitors to use the technique	☐	☐	☐	☐
5. ***Home visitors*** **support and encourage** one another to use the technique	☐	☐	☐	☐
6. Supervisors or other program staff **formally monitor and give feedback** on how well home visitors use the technique relative to program expectations	☐	☐	☐	☐

D) [DISPLAY IF 'Required' or 'Recommend' selected in C01-D]

Please indicate whether your program has any of the following implementation systems in place to support home visitors to use the technique "**Provide Social Support**"

Definition: Directly provide the pregnant woman encouragement, emotional support or practical help to perform a behavior.

	Yes, Fully in Place	Yes, Partially in Place	No	Not sure
1. Formal written policy clearly **defines when and how** to use the technique	☐	☐	☐	☐
2. Home visitors receive **formal training on when and how** to use the technique effectively	☐	☐	☐	☐
3. Home visitors receive **a formal assessment after completing training** to confirm they can use the technique effectively	☐	☐	☐	☐
4. ***Supervisors*** **support and encourage** home visitors to use the technique	☐	☐	☐	☐
5. ***Home visitors*** **support and encourage** one another to use the technique	☐	☐	☐	☐
6. Supervisors or other program staff **formally monitor and give feedback** on how well home visitors use the technique relative to program expectations	☐	☐	☐	☐

E) [DISPLAY IF 'Required' or 'Recommend' selected in C01-E]

Please indicate whether your program has any of the following implementation systems in place to support home visitors to use the technique "**Suggest or Arrange Social Support**"

Definition: Suggest or assist the pregnant woman to seek encouragement, emotional support or practical help to perform a behavior from a friend, relative, colleague, or group.

	Yes, Fully in Place	Yes, Partially in Place	No	Not sure
1. Formal written policy clearly **defines when and how** to use the technique	☐	☐	☐	☐
2. Home visitors receive **formal training on when and how** to use the technique effectively	☐	☐	☐	☐
3. Home visitors receive **a formal assessment after completing training** to confirm they can use the technique effectively	☐	☐	☐	☐
4. ***Supervisors*** **support and encourage** home visitors to use the technique	☐	☐	☐	☐
5. ***Home visitors*** **support and encourage** one another to use the technique	☐	☐	☐	☐
6. Supervisors or other program staff **formally monitor and give feedback** on how well home visitors use the technique relative to program expectations	☐	☐	☐	☐

F) [DISPLAY IF 'Required' or 'Recommend' selected in C01-F]

Please indicate whether your program has any of the following implementation systems in place to support home visitors to use the technique "**Natural Consequences**"

Definition: Provide written, verbal or visual information about a behavior's health, emotional, social or environmental consequences; encourage her to assess her feelings after attempts to perform a wanted behavior; raise her awareness of future regret about performing an unwanted behavior.

Note: This technique includes multiple strategies. If your program has different levels of implementation systems for strategies within the technique, select the response that reflects the highest level among the strategies.

	**Yes, Fully in Place**	**Yes, Partially in Place**	**No**	**Not sure**
1. Formal written policy clearly **defines when and how** to use the technique	☐	☐	☐	☐
2. Home visitors receive **formal training on when and how** to use the technique effectively	☐	☐	☐	☐
3. Home visitors receive **a formal assessment after completing training** to confirm they can use the technique effectively	☐	☐	☐	☐
4. ***Supervisors*** **support and encourage** home visitors to use the technique	☐	☐	☐	☐
5. ***Home visitors*** **support and encourage** one another to use the technique	☐	☐	☐	☐
6. Supervisors or other program staff **formally monitor and give feedback** on how well home visitors use the technique relative to program expectations	☐	☐	☐	☐

G) [DISPLAY IF 'Required' or 'Recommend' selected in C01-G]

Please indicate whether your program has any of the following implementation systems in place to support home visitors to use the technique "**Shape Knowledge of Behavior**"

Definition: Provide information or instruction to shape the pregnant woman's knowledge of how to perform a behavior. This includes identification of behavioral 'triggers' and their perceived causes. 'Triggers' are thoughts or situations that lead to performance of the unwanted behavior.

	**Yes, Fully in Place**	**Yes, Partially in Place**	**No**	**Not sure**
1. Formal written policy clearly **defines when and how** to use the technique	☐	☐	☐	☐
2. Home visitors receive **formal training on when and how** to use the technique effectively	☐	☐	☐	☐
3. Home visitors receive **a formal assessment after completing training** to confirm they can use the technique effectively	☐	☐	☐	☐

4. ***Supervisors*** **support and encourage** home visitors to use the technique	☐	☐	☐	☐
5. ***Home visitors*** **support and encourage** one another to use the technique	☐	☐	☐	☐
6. Supervisors or other program staff **formally monitor and give feedback** on how well home visitors use the technique relative to program expectations	☐	☐	☐	☐

H) [DISPLAY IF 'Required' or 'Recommend' selected in C01-H]

Please indicate whether your program has any of the following implementation systems in place to support home visitors to use the technique "**Antecedents**"

Definition: Change or support change of the pregnant woman's physical or social surroundings to facilitate performing a wanted behavior, create barriers to an unwanted behavior, or avoid cues to an unwanted behavior.

Note: This technique includes multiple strategies. If your program has different levels of implementation systems for strategies within the technique, select the response that reflects the highest level among the strategies.

	**Yes, Fully in Place**	**Yes, Partially in Place**	**No**	**Not sure**
1. Formal written policy clearly **defines when and how** to use the technique	☐	☐	☐	☐
2. Home visitors receive **formal training on when and how** to use the technique effectively	☐	☐	☐	☐
3. Home visitors receive **a formal assessment after completing training** to confirm they can use the technique effectively	☐	☐	☐	☐
4. ***Supervisors*** **support and encourage** home visitors to use the technique	☐	☐	☐	☐
5. ***Home visitors*** **support and encourage** one another to use the technique	☐	☐	☐	☐
6. Supervisors or other program staff **formally monitor and give feedback** on how well home visitors use the technique relative to program expectations	☐	☐	☐	☐

I) [DISPLAY IF 'Required' or 'Recommend' selected in C01-I]

Please indicate whether your program has any of the following implementation systems in place to support home visitors to use the technique "**Behavior Observation**"

Definition: Demonstrate a behavior; provide an observable example of a behavior; draw attention to others' performance of a behavior as a model.

Note: This technique includes multiple strategies. If your program has different levels of implementation systems for strategies within the technique, select the response that reflects the highest level among the strategies.

	Yes, Fully in Place	Yes, Partially in Place	No	Not sure
1. Formal written policy clearly **defines when and how** to use the technique	☐	☐	☐	☐
2. Home visitors receive **formal training on when and how** to use the technique effectively	☐	☐	☐	☐
3. Home visitors receive **a formal assessment after completing training** to confirm they can use the technique effectively	☐	☐	☐	☐
4. ***Supervisors*** **support and encourage** home visitors to use the technique	☐	☐	☐	☐
5. ***Home visitors*** **support and encourage** one another to use the technique	☐	☐	☐	☐
6. Supervisors or other program staff **formally monitor and give feedback** on how well home visitors use the technique relative to program expectations	☐	☐	☐	☐

J) [DISPLAY IF 'Required' or 'Recommend' selected in C01-J]

Please indicate whether your program has any of the following implementation systems in place to support home visitors to use the technique "**Associations to Promote Wanted Behavior**"

Definition: Identify, introduce, or alter social or environmental prompts or cues to promote a wanted behavior.

	Yes, Fully in Place	Yes, Partially in Place	No	Not sure
1. Formal written policy clearly **defines when and how** to use the technique	☐	☐	☐	☐
2. Home visitors receive **formal training on when and how** to use the technique effectively	☐	☐	☐	☐
3. Home visitors receive **a formal assessment after completing training** to confirm they can use the technique effectively	☐	☐	☐	☐
4. ***Supervisors*** **support and encourage** home visitors to use the technique	☐	☐	☐	☐
5. ***Home visitors*** **support and encourage** one another to use the technique	☐	☐	☐	☐
6. Supervisors or other program staff **formally monitor and give feedback** on how well home visitors use the technique relative to program expectations	☐	☐	☐	☐

K) [DISPLAY IF 'Required' or 'Recommend' selected in C01-K]

Please indicate whether your program has any of the following implementation systems in place to support home visitors to use the technique "**Associations to Deter Unwanted Behavior**"

Definition: Identify, alter, or remove social or environmental prompts or cues to deter an unwanted behavior.

	Yes, Fully in Place	Yes, Partially in Place	No	Not sure
1. Formal written policy clearly **defines when and how** to use the technique	☐	☐	☐	☐
2. Home visitors receive **formal training on when and how** to use the technique effectively	☐	☐	☐	☐
3. Home visitors receive **a formal assessment after completing training** to confirm they can use the technique effectively	☐	☐	☐	☐
4. ***Supervisors*** **support and encourage** home visitors to use the technique	☐	☐	☐	☐
5. ***Home visitors*** **support and encourage** one another to use the technique	☐	☐	☐	☐
6. Supervisors or other program staff **formally monitor and give feedback** on how well home visitors use the technique relative to program expectations	☐	☐	☐	☐

L) [DISPLAY IF 'Required' or 'Recommend' selected in C01-L]

Please indicate whether your program has any of the following implementation systems in place to support home visitors to use the technique "**Repetition & Substitution**"

Definition: Encourage the pregnant woman to practice performing a wanted behavior or substitute it for an unwanted behavior.

	Yes, Fully in Place	Yes, Partially in Place	No	Not sure
1. Formal written policy clearly **defines when and how** to use the technique	☐	☐	☐	☐
2. Home visitors receive **formal training on when and how** to use the technique effectively	☐	☐	☐	☐
3. Home visitors receive **a formal assessment after completing training** to confirm they can use the technique effectively	☐	☐	☐	☐
4. ***Supervisors*** **support and encourage** home visitors to use the technique	☐	☐	☐	☐
5. ***Home visitors*** **support and encourage** one another to use the technique	☐	☐	☐	☐
6. Supervisors or other program staff **formally monitor and give feedback** on how well home visitors use the technique relative to program expectations	☐	☐	☐	☐

M) [DISPLAY IF 'Required' or 'Recommend' selected in C01-M]

Please indicate whether your program has any of the following implementation systems in place to support home visitors to use the technique "**Comparison of Outcomes**"

Definition: Encourage the pregnant woman to compare the pros and cons of changing a behavior, or to compare the outcomes of changing versus not changing a behavior. Includes encouraging the pregnant woman's imagination or observation of either the consequences of an unwanted behavior or rewards for a wanted behavior.

	Yes, Fully in Place	Yes, Partially in Place	No	Not sure
1. Formal written policy clearly **defines when and how** to use the technique	☐	☐	☐	☐
2. Home visitors receive **formal training on when and how** to use the technique effectively	☐	☐	☐	☐
3. Home visitors receive **a formal assessment after completing training** to confirm they can use the technique effectively	☐	☐	☐	☐
4. ***Supervisors*** **support and encourage** home visitors to use the technique	☐	☐	☐	☐
5. ***Home visitors*** **support and encourage** one another to use the technique	☐	☐	☐	☐
6. Supervisors or other program staff **formally monitor and give feedback** on how well home visitors use the technique relative to program expectations	☐	☐	☐	☐

N) [DISPLAY IF 'Required' or 'Recommend' selected in C01-N]

Please indicate whether your program has any of the following implementation systems in place to support home visitors to use the technique "**Credible Source**"

Definition: Present verbal or visual communication from a credible source in favor of or against a behavior.

	Yes, Fully in Place	Yes, Partially in Place	No	Not sure
1. Formal written policy clearly **defines when and how** to use the technique	☐	☐	☐	☐
2. Home visitors receive **formal training on when and how** to use the technique effectively	☐	☐	☐	☐
3. Home visitors receive **a formal assessment after completing training** to confirm they can use the technique effectively	☐	☐	☐	☐
4. ***Supervisors*** **support and encourage** home visitors to use the technique	☐	☐	☐	☐
5. ***Home visitors*** **support and encourage** one another to use the technique	☐	☐	☐	☐

6. Supervisors or other program staff **<u>formally monitor and give feedback</u>** on how well home visitors use the technique relative to program expectations	☐	☐	☐	☐

O) [DISPLAY IF ‘Required’ or ‘Recommend’ selected in C01-O]

Please indicate whether your program has any of the following implementation systems in place to support home visitors to use the technique “**Incentives & Rewards**”

Definition: Provide or arrange for the pregnant woman to receive a material incentive or reward (something of value) or a social incentive or reward (words of congratulation), or removal of an unpleasant consequence for making progress in performing a behavior. Includes encouraging the pregnant woman to use self-incentives or self-rewards.

	**Yes, Fully in Place**	**Yes, Partially in Place**	**No**	**Not sure**
1. Formal written policy clearly **<u>defines when and how</u>** to use the technique	☐	☐	☐	☐
2. Home visitors receive **<u>formal training on when and how</u>** to use the technique effectively	☐	☐	☐	☐
3. Home visitors receive **<u>a formal assessment after completing training</u>** to confirm they can use the technique effectively	☐	☐	☐	☐
4. ***Supervisors*** **<u>support and encourage</u>** home visitors to use the technique	☐	☐	☐	☐
5. ***Home visitors*** **<u>support and encourage</u>** one another to use the technique	☐	☐	☐	☐
6. Supervisors or other program staff **<u>formally monitor and give feedback</u>** on how well home visitors use the technique relative to program expectations	☐	☐	☐	☐

P) [DISPLAY IF ‘Required’ or ‘Recommend’ selected in C01-P]

Please indicate whether your program has any of the following implementation systems in place to support home visitors to use the technique “**Scheduled Consequences**”

Definition: Use a threat of future punishment or removal of a reward as a consequence of performance of an unwanted behavior; arrange for a negative consequence or punishment following performance of an unwanted behavior.

<u>Note</u>: This technique includes multiple strategies. If your program has different levels of implementation systems for strategies within the technique, select the response that reflects the highest level among the strategies.

	Yes, Fully in Place	Yes, Partially in Place	No	Not sure
1. Formal written policy clearly **defines when and how** to use the technique	☐	☐	☐	☐
2. Home visitors receive **formal training on when and how** to use the technique effectively	☐	☐	☐	☐
3. Home visitors receive **a formal assessment after completing training** to confirm they can use the technique effectively	☐	☐	☐	☐
4. ***Supervisors*** **support and encourage** home visitors to use the technique	☐	☐	☐	☐
5. ***Home visitors*** **support and encourage** one another to use the technique	☐	☐	☐	☐
6. Supervisors or other program staff **formally monitor and give feedback** on how well home visitors use the technique relative to program expectations	☐	☐	☐	☐

Q) [DISPLAY IF 'Required' or 'Recommend' selected in C01-Q]

Please indicate whether your program has any of the following implementation systems in place to support home visitors to use the technique "**Mental Regulation**"

Definition: Suggest strategies to minimize demands on the pregnant woman's mental resources to make it easier for her to perform a wanted behavior.

	Yes, Fully in Place	Yes, Partially in Place	No	Not sure
1. Formal written policy clearly **defines when and how** to use the technique	☐	☐	☐	☐
2. Home visitors receive **formal training on when and how** to use the technique effectively	☐	☐	☐	☐
3. Home visitors receive **a formal assessment after completing training** to confirm they can use the technique effectively	☐	☐	☐	☐
4. ***Supervisors*** **support and encourage** home visitors to use the technique	☐	☐	☐	☐
5. ***Home visitors*** **support and encourage** one another to use the technique	☐	☐	☐	☐
6. Supervisors or other program staff **formally monitor and give feedback** on how well home visitors use the technique relative to program expectations	☐	☐	☐	☐

R) [DISPLAY IF 'Required' or 'Recommend' selected in C01-R]

Please indicate whether your program has any of the following implementation systems in place to support home visitors to use the technique "**Identity as Example to Others**"

Definition: Suggest to the pregnant woman that performing a wanted behavior might serve as an example to others.

	Yes, Fully in Place	Yes, Partially in Place	No	Not sure
1. Formal written policy clearly **defines when and how** to use the technique	☐	☐	☐	☐
2. Home visitors receive **formal training on when and how** to use the technique effectively	☐	☐	☐	☐
3. Home visitors receive **a formal assessment after completing training** to confirm they can use the technique effectively	☐	☐	☐	☐
4. ***Supervisors*** **support and encourage** home visitors to use the technique	☐	☐	☐	☐
5. ***Home visitors*** **support and encourage** one another to use the technique	☐	☐	☐	☐
6. Supervisors or other program staff **formally monitor and give feedback** on how well home visitors use the technique relative to program expectations	☐	☐	☐	☐

S) [DISPLAY IF ‘Required’ or ‘Recommend’ selected in C01-S]

Please indicate whether your program has any of the following implementation systems in place to support home visitors to use the technique “**Self-identity**”

Definition: Assist the pregnant woman to identify discrepancies between her behavior and her values or self-image; encourage her to self-identify as someone who used to perform an unwanted behavior; suggest her adopting a new perspective to change thoughts or emotions about a behavior.

Note: This technique includes multiple strategies. If your program has different levels of implementation systems for strategies within the technique, select the response that reflects the highest level among the strategies.

	Yes, Fully in Place	Yes, Partially in Place	No	Not sure
1. Formal written policy clearly **defines when and how** to use the technique	☐	☐	☐	☐
2. Home visitors receive **formal training on when and how** to use the technique effectively	☐	☐	☐	☐
3. Home visitors receive **a formal assessment after completing training** to confirm they can use the technique effectively	☐	☐	☐	☐
4. ***Supervisors*** **support and encourage** home visitors to use the technique	☐	☐	☐	☐
5. ***Home visitors*** **support and encourage** one another to use the technique	☐	☐	☐	☐
6. Supervisors or other program staff **formally monitor and give feedback** on how well home	☐	☐	☐	☐

visitors use the technique relative to program expectations

T) [DISPLAY IF 'Required' or 'Recommend' selected in C01-T]

Please indicate whether your program has any of the following implementation systems in place to support home visitors to use the technique "**Self-belief**"

Definition: Promote the pregnant woman's self-belief that she can successfully perform a behavior, for example by persuading her about her capabilities and encouraging her to mentally rehearse success, focus on past success or use positive self-talk.

	Yes, Fully in Place	Yes, Partially in Place	No	Not sure
1. Formal written policy clearly **defines when and how** to use the technique	☐	☐	☐	☐
2. Home visitors receive **formal training on when and how** to use the technique effectively	☐	☐	☐	☐
3. Home visitors receive **a formal assessment after completing training** to confirm they can use the technique effectively	☐	☐	☐	☐
4. ***Supervisors*** **support and encourage** home visitors to use the technique	☐	☐	☐	☐
5. ***Home visitors*** **support and encourage** one another to use the technique	☐	☐	☐	☐
6. Supervisors or other program staff **formally monitor and give feedback** on how well home visitors use the technique relative to program expectations	☐	☐	☐	☐

U) [DISPLAY IF 'Required' or 'Recommend' selected in C01-U]

Please indicate whether your program has any of the following implementation systems in place to support home visitors to use the technique "**Referral & Linkage**"

Definition: Provide referral or information to link the pregnant woman to a community resource to assist in performing a behavior; review progress in completing the referral; support connections in completing the referral or perform an interagency case review.

Note: This technique includes multiple strategies. If your program has different levels of implementation systems for strategies within the technique, select the response that reflects the highest level among the strategies.

	Yes, Fully in Place	Yes, Partially in Place	No	Not sure
1. Formal written policy clearly **defines when and how** to use the technique	☐	☐	☐	☐
2. Home visitors receive **formal training on when and how** to use the technique effectively	☐	☐	☐	☐

3. Home visitors receive **a formal assessment after completing training** to confirm they can use the technique effectively	☐	☐	☐	☐
4. ***Supervisors*** **support and encourage** home visitors to use the technique	☐	☐	☐	☐
5. ***Home visitors*** **support and encourage** one another to use the technique	☐	☐	☐	☐
6. Supervisors or other program staff **formally monitor and give feedback** on how well home visitors use the technique relative to program expectations	☐	☐	☐	☐

V) [DISPLAY IF 'Required' or 'Recommend' selected in C01-V]

Please indicate whether your program has any of the following implementation systems in place to support home visitors to use the technique "**Monitoring & Follow-up of Referral**"

Definition: Review the pregnant woman's experience accessing community resources to help her perform a behavior; assist in overcoming barriers to completing a referral.

Note: This technique includes multiple strategies. If your program has different levels of implementation systems for strategies within the technique, select the response that reflects the highest level among the strategies.

	**Yes, Fully in Place**	**Yes, Partially in Place**	**No**	**Not sure**
1. Formal written policy clearly **defines when and how** to use the technique	☐	☐	☐	☐
2. Home visitors receive **formal training on when and how** to use the technique effectively	☐	☐	☐	☐
3. Home visitors receive **a formal assessment after completing training** to confirm they can use the technique effectively	☐	☐	☐	☐
4. ***Supervisors*** **support and encourage** home visitors to use the technique	☐	☐	☐	☐
5. ***Home visitors*** **support and encourage** one another to use the technique	☐	☐	☐	☐
6. Supervisors or other program staff **formally monitor and give feedback** on how well home visitors use the technique relative to program expectations	☐	☐	☐	☐

W) [DISPLAY IF 'Required' or 'Recommend' selected in C01-W]

Please indicate whether your program has any of the following implementation systems in place to support home visitors to use the technique "**Coordination with Other Services**"

Definition: Ask about and act on the pregnant woman's ideas on how to assist her in adhering to guidance from other providers regarding performing a behavior.

	Yes, Fully in Place	Yes, Partially in Place	No	Not sure
1. Formal written policy clearly **defines when and how** to use the technique	☐	☐	☐	☐
2. Home visitors receive **formal training on when and how** to use the technique effectively	☐	☐	☐	☐
3. Home visitors receive **a formal assessment after completing training** to confirm they can use the technique effectively	☐	☐	☐	☐
4. ***Supervisors*** **support and encourage** home visitors to use the technique	☐	☐	☐	☐
5. ***Home visitors*** **support and encourage** one another to use the technique	☐	☐	☐	☐
6. Supervisors or other program staff **formally monitor and give feedback** on how well home visitors use the technique relative to program expectations	☐	☐	☐	☐

SECTION D: CONTACT AND PAYMENT

Thank you for completing this survey!

We would like to give you a $50 gift card for your time. Please provide the following information so that we can send you a Target e-gift card.

D01. Your Name (first and last): [TEXT BOX] ____________________
D02. Name of your program: [TEXT BOX] _____________________
D03. Your email: [TEXT BOX] ____________________

THANK YOU AGAIN FOR TAKING PART IN THIS SURVEY AND FOR ALL YOU DO TO HELP FAMILIES!

Additional File 1 – Survey as distributed to local program representatives
