# Supplementary material for: Moving toward precision in prenatal evidence-based home visiting to achieve good birth outcomes: assessing the alignment of local programs with their national models
Source: BMC Health Serv Res. 2023 Jul 29;23:812. doi: 10.1186/s12913-023-09815-8 (PMC10386274; doi:10.1186/s12913-023-09815-8)
Supplement: Supplementary file 2 — Additional file 2. [file 12913_2023_9815_MOESM2_ESM.pdf]

1.	**Assess Readiness for Change** Gather information about the expectant woman's readiness to change the behavior.
2.	**Goals & Planning** Assist the expectant woman to: set a behavior change goal; develop a plan to meet the goal using strategies to overcome barriers and increase facilitators; review her progress toward the goal; modify the goal or plan as needed.
3.	**Monitoring & Feedback** Monitor the expectant woman's progress in changing the behavior; give feedback on that progress; establish ways for the expectant woman to self-monitor her progress.
4.	**Provide Social Support** Directly provide the expectant woman encouragement, emotional support or practical help to perform the behavior.
5.	**Suggest or Arrange Social Support** Suggest or assist the expectant woman to seek encouragement, emotional support or practical help to perform the behavior from a friend, relative, colleague, or group.
6.	**Natural Consequences** Provide written, verbal or visual information about the behavior's health, emotional, social or environmental consequences; encourage her to assess her feelings after attempts to perform the behavior; raise her awareness of future regret about performing the unwanted behavior.
7.	**Shape Knowledge of Behavior** Provide information or instruction to shape the expectant woman's knowledge of *how to perform the behavior*. This includes identification of behavioral 'triggers' and their perceived causes. 'Triggers' are thoughts or situations that lead to performance of the unwanted behavior.
8.	**Antecedents** Change or support change of the expectant woman's physical or social surroundings to facilitate performing the behavior, create barriers to an unwanted behavior, or avoid cues to an unwanted behavior.
9.	**Behavior Observation** Demonstrate the behavior; provide an observable example of the behavior; draw attention to others' performance of the behavior as a model.
10.	**Associations to Promote Wanted Behavior** Identify, introduce, or alter social or environmental prompts or cues to promote the wanted behavior.
11.	**Associations to Deter Unwanted Behavior** Identify, alter, or remove social or environmental prompts or cues to deter the unwanted behavior.
12.	**Repetition & Substitution** Encourage the expectant woman to practice performing the behavior or substitute it for an unwanted behavior.
13.	**Comparison of Outcomes** Encourage the expectant woman to compare the pros and cons of changing the behavior, or to compare the outcomes of changing versus not changing the behavior. Includes encouraging the expectant woman's imagination or observation of either the consequences of the unwanted behavior or rewards for the wanted behavior.
14.	**Credible Source** Present verbal or visual communication from a credible source in favor of or against the behavior.
15.	**Incentives & Rewards** Provide or arrange for the expectant woman to receive a *material incentive or reward* (something of value) or a *social incentive or reward* (words of congratulation), or removal of an unpleasant consequence for making progress in performing the behavior. Includes encouraging the expectant woman to use self-incentives or self-rewards.
16.	**Scheduled Consequences** Use a threat of future punishment or removal of a reward as a consequence of performance of an unwanted behavior; arrange for a negative consequence or punishment following performance of an unwanted behavior.
17.	**Mental Regulation** Suggest strategies to minimize demands on the expectant woman's mental resources to make it easier for her to perform the behavior.
18.	**Identity as Example to Others** Suggest to the expectant woman that performing the behavior might serve as an example to others.
19.	**Self-identity** Assist the expectant woman to identify discrepancies between her behavior and her values or self-image; encourage her to self-identify as someone who *used to* perform the unwanted behavior; suggest her adopting a new perspective to change thoughts or emotions about the behavior.
20.	**Self-belief** Promote the expectant woman's self-belief that she can successfully perform the behavior, for example by persuading her about her capabilities and encouraging her to mentally rehearse success, focus on past success or use positive self-talk.
21.	**Referral & Linkage** Provide referral or information to link the expectant woman to a community resource to assist in performing the behavior; review progress in completing the referral; support connections in completing the referral or perform an interagency case review.
22.	**Monitoring & Follow-up of Referral** Review the expectant woman's experience accessing community resources to help her perform the behavior; assist in overcoming barriers to completing a referral.
23.	**Coordination with Other Services** Ask about and act on the expectant woman's ideas on how to assist her in adhering to guidance from other providers regarding performing the behavior.
